# An Adaptive Moving Mesh Method for Forced Curve Shortening Flow*

**DOI:** 10.1137/18M1211969

**Published:** 2019

**Authors:** J. A. Mackenzie, M. Nolan, C. F. Rowlatt, R. H. Insall

**Affiliations:** ‡ Department of Mathematics and Statistics, University of Strathclyde, Glasgow G1 1XH, UK; § Cancer Research UK Beatson Institute, Glasgow G61 1BD, UK

**Keywords:** geometric partial differential equations, forced curve shortening flow, moving mesh methods, tangential redistribution, monitor functions, 35K65, 53C44, 53C80, 65M06, 65M50

## Abstract

We propose a novel adaptive moving mesh method for the numerical solution of a forced curve shortening geometric evolution equation. Control of the mesh quality is obtained using a tangential mesh velocity derived from a mesh equidistribution principle, where a positive adaptivity measure or monitor function is approximately equidistributed along the evolving curve. Central finite differences are used to discretize in space the governing evolution equation for the position vector, and a second-order implicit scheme is used for the temporal integration. Simulations are presented indicating the generation of meshes which resolve areas of high curvature and are of second-order accuracy. Furthermore, the new method delivers improved solution accuracy compared to the use of uniform arc-length meshes.

## 1 Introduction

Within the past 20 years there has been much interest in the numerical approximation of geometric flows (see, for example, [12, 14]). In this paper we consider an adaptive method for the solution of the curve evolution equation

(1.1) $$V\left(x,t\right):=\dot{\text{x}}\cdot \text{n}=\alpha \left(\text{x},t\right)\kappa +\beta \left(\text{x},t\right),$$

where ***x*** is the position vector of the evolving curve Γ, *α* and *β* are given functions with *α* being nonnegative, and *κ* is the curvature. In the special case when *α* (***x**, t*) = 1 and *β* (***x**, t*) = 0 we get classical curve shortening flow. Geometric equations of the form (1.1) appear in many important application areas, such as material science [1], biological cell migration [29, 16], and image processing [20].

The numerical solution of geometric evolution laws poses many challenges, and a number of different techniques have been proposed which fall broadly into two categories: embedded methods and sharp interface methods. Examples of embedded techniques include phase-field methods [12] and the level set method [32, 30]. These methods identify the moving interface as the zero level set of an indicator function which is normally evolved through a fixed uniform background mesh. Grid generation is therefore not an issue, although in reality efficient implementations of embedded methods may require some form of mesh adaptation.

Sharp interface or interface tracking methods represent the curve by the positions of a discrete set of nodal positions on the curve, and these points are evolved in such a way that their normal velocity satisfies (1.1). It is well appreciated, however, that methods which move mesh nodes purely in the normal direction quickly run into difficulty due to over concentration of grid points in areas with locally converging normals and to the opposite problem of dispersion of grid points in areas with diverging local normals. This can lead to lower accuracy, grid crossover, and instabilities, all of which can only be avoided by using an unreasonably small time step.

One way to maintain a good mesh quality is to introduce a tangential velocity ***ℬ*** so that mesh nodes evolve according to the equation

(1.2) $$\dot{\text{x}}=V\text{n}+\text{ℬt}.$$

This approach is attractive because the presence of a tangential velocity has no effect on the shape of the evolving curve, as the shape is determined purely by its normal velocity *𝒱*. Many suggestions have been made of a suitable tangential velocity to improve solution accuracy and robustness. A nonlocal choice of ***ℬ*** originally proposed by Hou, Lowengrub, and Shelley [17] maintains the relative local curve length between grid points. In [25] this method was generalized so that mesh points evolve to asymptotically equidistribute the arc-length between grid nodes. An alternative approach, giving rise to an intrinsic tangential velocity, was proposed by Barrett, Garcke, and Nürnberg (BGN) in a series of papers [3, 4]. Their method was shown to produce good quality meshes for a range of geometric evolution laws for curves in ℝ^2^ and hypersurfaces in ℝ^3^. The fully discrete original BGN schemes use a semi-implicit temporal integration method and hence are not guaranteed to exactly equidistribute arc-length. A fully implicit version of the BGN scheme was later proposed which exactly equidistributes arc-length [5]. However, exact equidistribution comes at the cost of having to solve a nonlinear system of equations at each time step. More recently, Elliott and Fritz proposed a finite element method using the DeTurk trick for curve shortening and mean curvature flow [15]. This method involves a parameter which interpolates between the methods of BGN and the scheme of Deckelnick [11]. The parameter also controls the rate at which grid nodes evolve to equidistribute arc-length.

All of the fully discrete BGN schemes and the Elliott and Fritz scheme are first-order accurate in time. Second-order temporal accuracy is achieved using a Crank–Nicolson scheme in [2], and the simulations presented there suggest that a considerable improvement in accuracy can be obtained using a higher-order time integration scheme. Solution accuracy can also be improved using some form of adaptive meshing technique because areas of high curvature require additional local resolution, and this cannot be achieved using a uniform arc-length mesh.

For time-dependent PDEs with localized solution features the use of adaptive moving mesh methods has proved popular [8, 19]. These methods generally use a fixed number of mesh nodes which are redistributed at each time step. Recently, we introduced an adaptive moving mesh method for the evolution of a curve which is driven in the normal direction by a function of curvature and a forcing function. In the tangential direction mesh points are moved according to a moving mesh PDE (MMPDE). The adaptive moving curve method forms part of a fitted bulk-surface formulation of a model of cell migration [21]. The aim of this paper is to improve and extend the method introduced in [21]. The equation for the tangential velocity is derived within the context of a gradient flow equation for the minimization of a functional related to the equidistribution of a mesh adaptivity criterion or monitor function. Within this class of methods, specific choices of the gradient flow direction are shown to reproduce some methods in the literature. To drive mesh adaptivity we develop a monitor function based on curvature. We present two temporal discretizations of the moving mesh equations and show that a newly proposed method is second-order accurate in time and space.

An outline of the rest of this paper is as follows. In the next section we present the geometric evolution law for the curve normal velocity as well as the tangential velocity arising from an adaptive moving mesh approach. In this section we also derive a monitor function to drive the tangential motion of mesh points to areas of high curvature to improve solution accuracy. The numerical discretization of the curve evolution equations is given in section 3. Numerical experiments are carried out in section 4 highlighting the improved performance of the moving mesh method compared to a uniform arc-length redistribution of mesh points. Finally, we make some conclusions and point out directions for future research in section 5.

## 2 Forced curve shortening flow

A closed, embedded, regular plane curve Γ(*t*) can be parameterized by the smooth function ***x***(*t*) : ℝ/ℤ ⊃ [0, 1] → ℝ^2^, such that Γ(*t*) = Image(***x***(*t*)) := {***x***(*ξ*, *t*), *ξ* ∈ [0, 1]}. Let *F*_*ξ*_ = ∂*F*/∂*ξ* and $\left|\text{a}\right|=\sqrt{\text{a}\cdot \text{a}},$ where ***a*** · ***b*** denotes the Euclidean inner product between the vectors ***a*** and ***b***. The unit tangent vector ***t*** = ***x****_ξ_*/|***x****_ξ_*| = ***x**_s_*, where *s* is the arc-length parameter and d*s* = |***x***_ξ_| d*ξ*. We define the unit normal vector ***n*** such that det(***t**, **n***) = 1 and define the signed curvature in the direction ***n*** by *κ*.

Using the Frenet–Serret formula, we have

(2.1) $${\text{x}}_{ss}={\text{t}}_{s}=\kappa \text{n}.$$

Applying the chain rule, we have

(2.2) $${\text{x}}_{\xi }={\text{x}}_{s}\frac{ds}{d\xi }={\text{x}}_{s}|{\text{x}}_{\xi }|,$$

and differentiation of (2.2) with respect to *ξ* and the use of (2.1) leads to the relation

(2.3) $${\text{x}}_{\xi \xi }={\text{x}}_{ss}{\left|{\text{x}}_{\xi }\right|}^{2}+{\text{x}}_{s}{\left|{\text{x}}_{\xi }\right|}_{s}\left|{\text{x}}_{\xi }\right|=\kappa {\left|{\text{x}}_{\xi }\right|}^{2}\text{n}+|{\text{x}}_{\xi }{|}_{\xi }\text{t}.$$

If we multiply through (2.3) by ***n***, we can therefore express the curvature

(2.4) $$\kappa =\frac{{\text{x}}_{\xi \xi }\cdot \text{n}}{{\left|{\text{x}}_{\xi }\right|}^{2}},$$

and hence in terms of the parameterization *ξ*, we can express the normal velocity as

(2.5) $$𝒱=\dot{\text{x}}\cdot n=\alpha (\text{x},t)\left(\frac{{\text{x}}_{\xi \xi }\cdot n}{{\left|{\text{x}}_{\xi }\right|}^{2}}\right)+\beta (\text{x},t).$$

### 2.1 Adaptive moving mesh approach for the tangential velocity

The proposed tangential mesh velocity is based on the idea of mesh equidistribution. Let *M* (***x**, t*) > 0 be a positive monitor function indicating areas of the curve which require additional resolution, such as regions of high curvature. The *ξ*-parameterization is said to equidistribute *M* over the curve Γ(*t*) if

(2.6) $$M\;\frac{ds}{d\xi }=\int_{\text{Γ}(t)}M\;\text{d}s.$$

Differentiating (2.6) with respect to *ξ* we have the equivalent equidistribution condition,

(2.7) $${\left(M\frac{ds}{d\xi }\right)}_{\xi }={\left(M\left|{\text{x}}_{\xi }\right|\right)}_{\xi }=0.$$

If the parametric domain is partitioned uniformly by grid points ${\left\{\xi_{i}\right\}}_{i=0}^{N},$ then the equidistribution condition (2.7) essentially ensures that the image mesh points on the curve are arranged so that the weighted arc-length *M* d*s* is constant. The derivation of a suitable curvature-based monitor function is given in section 2.2. If *M* is constant, then the satisfaction of the equidistribution condition leads to a uniform parameterization in terms of arc-length.

The equidistribution condition, in terms of the inverse mapping, is the Euler–Lagrange equation for the minimizer of the functional

(2.8) $$I(\xi (s,t))=\frac{1}{2}{\int_{\text{Γ}(t)}\frac{1}{M}\left(\frac{\partial \xi }{\partial s}\right)}^{2}\text{d}s.$$

An evolution equation can be obtained from the gradient flow equation

(2.9) $$\begin{array}{l} \frac{\partial \xi }{\partial t}=-\frac{P}{\tau }\frac{\delta I}{\delta s}\\ \;=\frac{P}{\tau }\frac{\partial }{\partial s}\left(\frac{1}{M}\frac{\partial \xi }{\partial s}\right). \end{array}$$

Here, *τ* > 0 is a mesh relaxation time determining the rate at which *ξ*(*s, t*) evolves to minimize (2.8). The positive definite differential operator *P* allows a degree of flexibility in the method as we show below. Equation (2.9) is not in an ideal form as the independent variable is arc-length, *s*, so we need to change the role of the independent and dependent variables. Starting from the identity

(2.10) ξ=ξ(s(ξ,t),t),

we can differentiate both sides with respect to *ξ* while keeping *t* fixed, and we find that

(2.11) $$\frac{\partial s}{\partial \xi }={\left(\frac{\partial \xi }{\partial s}\right)}^{-1}.$$

Differentiating (2.10) with respect to *t* while keeping *ξ* fixed gives

(2.12) $$\frac{\partial s}{\partial t}=-\frac{\partial s}{\partial \xi }\frac{\partial \xi }{\partial t}.$$

Equation (2.9) can therefore be rewritten as

(2.13) $$\frac{\partial s}{\partial t}=\frac{P}{\tau }{\left(M\frac{\partial s}{\partial \xi }\right)}^{-2}\frac{\partial }{\partial \xi }\left(M\frac{\partial s}{\partial \xi }\right).$$

Note that the time derivative of the position vector ***x*** in (1.2) is taken under the assumption that the value of the parameterization variable *ξ* is fixed. In terms of the arc-length parameterization, we have

(2.14) $$\begin{array}{c} \dot{x}\equiv {\frac{\partial x}{\partial t}|}_{\xi }={\frac{\partial x}{\partial t}|}_{s}+{\frac{\partial x}{\partial s}\frac{\partial s}{\partial t}|}_{\xi }\\ \;={\frac{\partial x}{\partial t}|}_{s}+{\frac{\partial s}{\partial t}|}_{\xi }t. \end{array}$$

Comparing (1.2) and (2.14) and using (2.13), we arrive at the tangential velocity equation

(2.15) $$ℬ=\dot{x}\cdot t=\frac{P}{\tau }{\left(M\left|x_{\xi }\right|\right)}^{-2}{\left(M\left|x_{\xi }\right|\right)}_{\xi }.$$

We can identify the equidistribution condition (2.7) as the driving force for tangential mesh movement, evolving the mesh nodes back towards the equidistribution of the monitor function *M* whenever it drifts away—the rate being controlled by the parameter *τ*. Particular choices for the operator *P* lead to distinct tangential velocities, some of which correlate with previously proposed methods. For example, attempting to minimize (2.9) by the steepest descent direction corresponds to the choice *P* = 1. In the special case when *M* = 1, *P* = 1, and *τ* = 1, the tangential velocity *ℬ* = –(|***x**_ξ_*|^−1^)_*ξ*_, which was used in [11] for curve shortening flow. The choice *P* = *M*|*x_ξ_*|^2^ results in the tangential velocity equation

(2.16) $$\dot{x}\cdot t=\frac{1}{M\tau }{\left(M\left|x_{\xi }\right|\right)}_{\xi }.$$

This choice of *P* results in a tangential velocity equation which is more spatially balanced throughout the domain [18]. In the particular case where a uniform arc-length mesh is desired (*M* = 1), (2.16) is identical to that used in a recent method proposed by Elliott and Fritz [15] based on a harmonic map heat flow.

### 2.2 Choice of monitor function

**Fig. 1:**
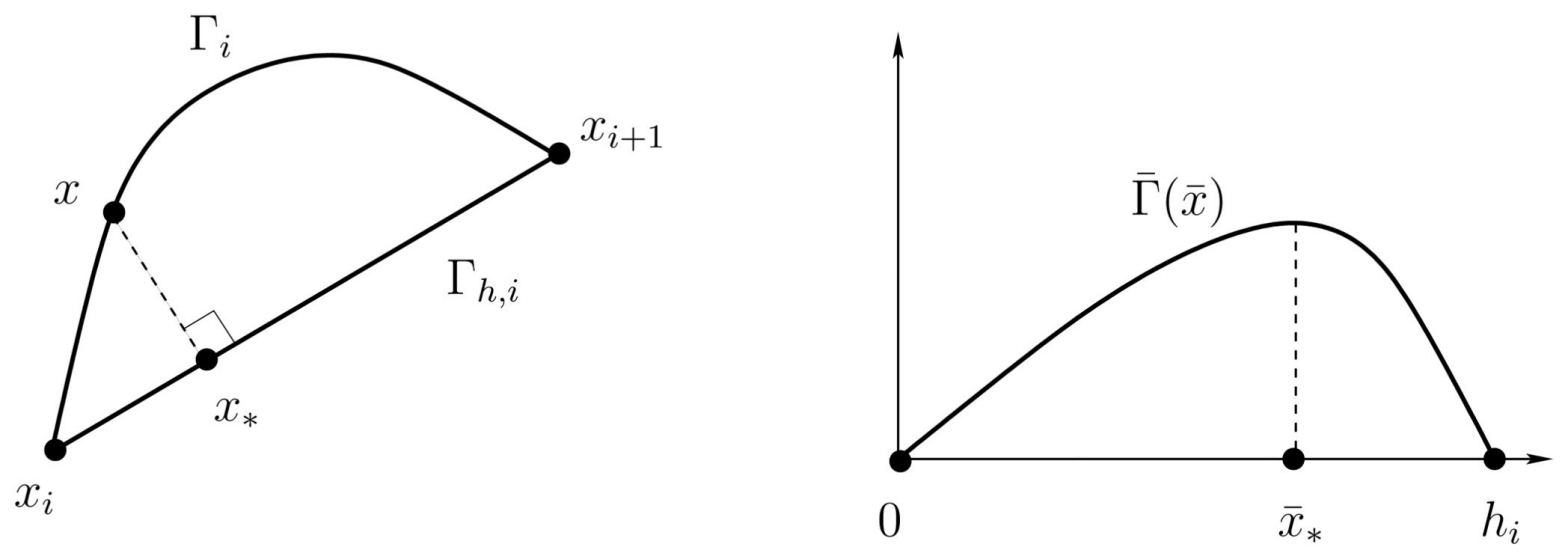
Left: Segment of a smooth curve Γ*_i_* and interpolating linear approximation Γ*_h,i_* between mesh points ***x**_i_* and ***x***_*i*+1_. The distance between the curves at point ***x*** is the distance from ***x*** to x_*_. Right: The translated and rotated segment is transformed into the graph $\bar{\text{Γ}}\left(\bar{x}\right).$ The maximal distance between Γ*_i_* and Γ*_h,i_* is equal to the absolute maximum value $\bar{\text{Γ}}\left({\bar{x}}_{*}\right).$

In the absence of a reliable error estimate for the approximation Γ_*h*_(*t*) of Γ(*t*), we base our analysis of a suitable monitor function on a study of interpolation error. The aim is to find a monitor function which, when equidistributed, results in a distribution of mesh points that minimizes an appropriate measure of the difference between a smooth curve Γ and its linear polygonal interpolant Γ*_h_*. Here we focus on the minimization of the maximal distance between Γ and Γ*_h_*. In Figure 1 we show the segment Γ*_i_* of Γ with end points ***x**_i_* and ***x***_*i*+1_. Also shown is the linear approximation Γ*_h,i_* of Γ*_i_*. For each ***x*** ∈ Γ*_i_*, we define the distance, *d*(***x***), from ***x*** to Γ*_h,i_* as the distance between ***x*** and x_*_ ∈ Γ*_h,i_*, where the line through ***x*** and x_*_ is perpendicular to Γ*_h,i_*. To simplify the analysis, we note that the distance between Γ*_i_* and Γ*_h,i_* is invariant to a coordinate rotation and translation. We therefore translate coordinates so that ***x**_i_* maps to the origin, and we rotate coordinates such that the line segment between ***x**_i_* and ***x**_i_*_+1_ is parallel to the positive $\bar{x}$ axis, as shown in Figure 1. Finding the maximal distance between Γ_*i*_ and Γ_*h,i*_ is therefore equivalent to finding the maximum absolute value of the transformed graph $\bar{\text{Γ}}\left(\bar{x}\right)$ for $0\leq \bar{x}\leq \left|x_{i+1}-x_{i}\right|,$ and this can be estimated using a standard argument from linear interpolation theory.

Without loss of generality, let us assume that the maximum of $\bar{\text{Γ}}$ occurs at $\bar{x}$_*_ and assume $\bar{x}$_*_ is closer to $\bar{x}$ = 0 than $\bar{x}=h_{i}\equiv \left|x_{i+1}-x_{i}\right|.$ Using a Taylor series expansion of $\bar{\text{Γ}}$ about $\bar{x}$ = 0, and noting that $\bar{\text{Γ}}$ (0) = 0 and ${\bar{\text{Γ}}}^{\prime }\left(\bar{\text{x}}*\right)=0,$ we have

$$\bar{\text{Γ}}({\bar{x}}_{*})=\frac{{\bar{x}}_{*}^{2}}{2}{\bar{\text{Γ}}}^{\prime \prime }({\bar{x}}_{*})+O{\left({\bar{x}}_{*}\right)}^{3}.$$

The absolute value of the curvature $\left|\bar{\kappa }\right|$ of $\bar{\text{Γ}}$ is

$$\left|\bar{\kappa }\right|=\frac{\left|{\bar{\text{Γ}}}^{\prime \prime }\right|}{{\left(1+{\left({\bar{\text{Γ}}}^{\prime }\right)}^{2}\right)}^{3/2}},$$

and since ${\bar{\text{Γ}}}^{\prime }\left({\bar{x}}_{*}\right)=0$, it follows that $\left|{\bar{\text{Γ}}}^{\prime \prime }\left({\bar{x}}_{*}\right)\right|=\left|\bar{\kappa }\left({\bar{x}}_{*}\right)\right|.$ Therefore, we find that

(2.17) $$\begin{array}{r} \underset{\bar{x}\in \left(0,h_{i}\right)}{\max}\left|\bar{\text{Γ}}\left(\bar{x}\right)\right|=\frac{{\bar{x}}_{*}^{2}}{2}\left|{\bar{\text{Γ}}}^{\prime \prime }\left({\bar{x}}_{*}\right)\right|+O{\left({\bar{x}}_{*}\right)}^{3}\\ \leq \frac{h_{i}^{2}}{8}\left|\bar{\kappa }\left({\bar{x}}_{*}\right)\right|+O{\left(h_{i}\right)}^{3}\text{.} \end{array}$$

The curvature of Γ_*i*_ is clearly invariant to the translation and rotation mapping above, and hence an approximately optimal distribution of mesh points ${\left\{{\text{x}}_{i}\right\}}_{i=0}^{N},$ which minimizes the maximal error over all segments, is obtained when

(2.18) $$\begin{array}{ll} h_{i}^{2}\left|\kappa_{i}\right|=h_{i+1}^{2}\left|\kappa_{i+1}\right|, & i=1,\ldots \end{array},N-1,$$

where *κ*_*i*_ = max_***x***∈Γ_*i*__ |*κ*(***x***)|. It therefore follows that the quantity *h_i_|κ_i_*|^1/2^ is constant in each segment, and this suggests that a suitable monitor function for curve approximation should be based on equidistribution of |*κ*|^1/2^.

Since *κ* can potentially be zero at flat sections of a curve, it is important to include a positive floor on the monitor function to ensure that no area of the curve becomes starved of mesh points. A simple curvature-based monitor function therefore takes the form

(2.19) $$M=\frac{1}{2}\left(M_{\text{floor}}+{\left|\kappa \right|}^{1/2}\right).$$

Motivated by the design of suitable monitor functions for singular perturbation problems [6], we consider the floor

(2.20) $$M_{\text{floor}}\left(t\right)=\frac{1}{\left|\text{Γ}\left(t\right)\right|}\int_{\text{Γ}\left(t\right)}{\left|\kappa \right|}^{1/2}\text{d}s\text{.}$$

A similar floor was used in the adaptive solution of evolutionary PDEs in one dimension [7]. Major advantages of the floor (2.20) are that it does not require any a priori choice of parameters and that it adapts to the length of the evolving curve.

Note that although we have focused on the derivation of a suitable monitor function based on the maximal distance between Γ*_h_*(*t*) and Γ(*t*), alternative monitor functions can be derived to minimize different error measures. For example, it has been shown that equidistribution of |*κ*|^1/3^ leads to an interpolatory linear polygonal curve which minimizes the discrepancy in the enclosed area between Γ and Γ_*h*_, and equidistribution of |*κ*|^2/3^ minimizes the total length discrepancy [33].

## 3 Numerical discretization

The time integration interval (0, *T*] is partitioned using *N_T_* time steps of size Δ*t* = *T/N_T_*. We represent the evolving curve at time *t^n^* = *n*Δ*t* by the closed linear polygonal curve joining the discrete plane points ${\text{x}}_{i}^{n},\;i=0,\ldots ,N.$ To enforce periodicity we set ${\text{x}}_{0}^{n}={\text{x}}_{N}^{n},$ and we also use the ghost nodes ${\text{x}}_{-1}^{n}={\text{x}}_{N-1}^{n}\;\text{and}\;{\text{x}}_{N+1}^{n}={\text{x}}_{1}^{n}.$ The parameterization interval *ξ* ∈ [0, 1] is discretized using a uniform step size Δ*ξ* = 1*/N*. Using central differences, we approximate the unit tangent vector at ${\text{x}}_{i}^{n}$ by

$${\text{t}}_{i}^{n}=\frac{{\text{x}}_{i+1}^{n}-{\text{x}}_{i-1}^{n}}{\left|{\text{x}}_{i+1}^{n}-{\text{x}}_{i-1}^{n}\right|}=\left(t_{1}^{n},t_{2}^{n}\right),$$

and we set ${\text{n}}_{i}^{n}=\left(t_{2}^{n},-t_{1}^{n}\right).$ We use central finite differences to approximate the spatial derivatives in (2.5), and we consider two temporal integration schemes. The first approach, which was introduced in [21], is based on a first-order fully implicit backward Euler scheme. This leads to a nonlinear algebraic system which is solved using Picard iteration. If ***x***^[*n,m*]^ denotes the approximation of ***x***^*n*^ at iteration level *m*, then the discretized normal velocity equation takes the form

(3.1) $$[-\mu_{i}^{\left[n+1,m\right]}{\text{x}}_{i-1}^{\left[n+1,m+1\right]}+(1+2\mu_{i}^{\left[n+1,m\right]}){\text{x}}_{i}^{\left[n+1,m+1\right]}-\mu_{i}^{\left[n+1,m\right]}{\text{x}}_{i+1}^{\left[n+1,m+1\right]}]\cdot {\text{n}}_{i}^{\left[n+1,m\right]}={\text{x}}_{i}^{n}\cdot {\text{n}}_{i}^{\left[n+1,m\right]}+\Delta t\beta_{i}^{\left[n+1,m\right]}$$

for *i* = 1, …, *N*, where

$$\begin{array}{l} \mu_{i}^{\left[n,m\right]}=\frac{4\Delta t\alpha_{i}^{\left[n,m\right]}}{{\left|{\text{x}}_{i+1}^{\left[n,m\right]}-{\text{x}}_{i-1}^{\left[n,m\right]}\right|}^{2}},\\ \alpha_{i}^{\left[n,m\right]}=\alpha \left({\text{x}}_{i}^{\left[n,m\right]},t^{n}\right)\;\text{and}\;\beta_{i}^{\left[n,m\right]}=\beta \left({\text{x}}_{i}^{\left[n,m\right]},t^{n}\right). \end{array}$$

The second scheme uses a second-order Crank–Nicolson fully implicit temporal discretization of the normal velocity equation (2.5), and the solution is found using the iteration

(3.2) $$\left[-\frac{\mu_{i}^{\left[n+1,m\right]}}{2}{\text{x}}_{i-1}^{\left[n+1,m+1\right]}+(1+\mu_{i}^{\left[n+1,m\right]}){\text{x}}_{i}^{\left[n+1,m+1\right]}-\frac{\mu_{i}^{\left[n+1,m\right]}}{2}{\text{x}}_{i+1}^{\left[n+1,m+1\right]}\right]\cdot {\text{n}}_{i}^{\left[n+\frac{1}{2},m\right]}=\frac{\mu_{i}^{n}}{2}[{\text{x}}_{i-1}^{n}-2{\text{x}}_{i}^{n}+{\text{x}}_{i+1}^{n}]\cdot {\text{n}}_{i}^{\left[n+\frac{1}{2},m\right]}+{\text{x}}_{i}^{n}\cdot {\text{n}}_{i}^{\left[n+\frac{1}{2},m\right]}+\frac{\Delta t}{2}(\beta_{i}^{\left[n+1,m\right]}+\beta_{i}^{n})$$

for *i* = 1, …, *N*, where

$${\text{n}}_{i}^{\left[n+\frac{1}{2},m\right]}=\frac{\left({\text{n}}_{i}^{\left[n+1,m\right]}+{\text{n}}_{i}^{n}\right)}{2}.$$

The spatial discretization is applied to a reformulation of the tangential velocity equation (2.15). Using the identity

$${\left|{\text{x}}_{\xi }\right|}_{\xi }=\frac{{\text{x}}_{\xi }\cdot {\text{x}}_{\xi \xi }}{\left|{\text{x}}_{\xi }\right|}$$

we can write (2.15) as

(3.3) $$\dot{x}\cdot \text{t}=\frac{P(M_{\xi }{\text{x}}_{\xi }+M{\text{x}}_{\xi \xi })\cdot \text{t}}{\tau {\left(M|{\text{x}}_{\xi }|\right)}^{2}}.$$

Since ***t*** = ***x***_*ξ*_/|***x***_*ξ*_|, we can rewrite (3.3) as

(3.4) $$\left(\dot{\text{x}}-\frac{PM}{\tau {\left(M\left|{\text{x}}_{\xi }\right|\right)}^{2}}{\text{x}}_{\xi \xi }\right)\cdot \text{t}=\frac{PM_{\xi }\left|{\text{x}}_{\xi }\right|}{\tau {\left(M\left|{\text{x}}_{\xi }\right|\right)}^{2}}.$$

To discretize (3.4) we use central differences to approximate the spatial terms and a first-order backward Euler time integration scheme. This results in the set of equations

(3.5) $$\left[-v_{i}^{\left[n+1,m\right]}{\text{x}}_{i-1}^{\left[n+1,m+1\right]}+(1+2v_{i}^{\left[n+1,m\right]}){\text{x}}_{i}^{\left[n+1,m+1\right]}-v_{i}^{\left[n+1,m\right]}{\text{x}}_{i+1}^{\left[n+1,m+1\right]}]\cdot {\text{t}}_{i}^{\left[n+1,m\right]}={\text{x}}_{i}^{n}\cdot {\text{t}}_{i}^{\left[n+1,m\right]}+\frac{\Delta tP_{i}^{n}(M_{i+1}^{n}-M_{i-1}^{n})}{\tau {\left(M_{i}^{n}\left|{\text{x}}_{i+1}^{\left[n+1,m\right]}-{\text{x}}_{i-1}^{\left[n+1,m\right]}\right|\right)}^{2}}(\left|{\text{x}}_{i+1}^{\left[n+1,m\right]}-{\text{x}}_{i-1}^{\left[n+1,m\right]}\right|\right)$$

for *i* = 1, …,*N*, where

$$v_{i}^{\left[n,m\right]}=\frac{4\Delta tM_{i}^{n}P_{i}^{n}}{\tau {\left(M_{i}^{n}\left|{\text{x}}_{i+1}^{\left[n+1,m\right]}-{\text{x}}_{i-1}^{\left[n+1,m\right]}\right|\right)}^{2}}.$$

Note that the monitor function *M* and spatial balancing operator *P* are always treated explicitly. This is justified because, in general, one may wish to adapt the mesh to solution features (such as a travelling wave front), which will only be known at time level *t^n^*. The coupled set of 2*N* equations, comprised of (3.1) or (3.2) for the normal velocity and (3.5) for the tangential velocity, is solved for ***x***^[*n*+1,*m*+1]^, and the Picard iteration is stopped when

$$\left|{\text{x}}^{\left[n+1,m+1\right]}-{\text{x}}^{\left[n+1,m\right]}\right|<10^{-6}.$$

The solution after the final iteration is then used as the approximation ***x***^*n*+1^. For the initial guess at the start of each iteration we set ***x***^[*n*+1,0]^ = ***x***^*n*^. The maximum number of iterations allowed is fixed at 200, and if the Picard solver cannot converge within this limit, then the simulation stops.

The first integration scheme is therefore a backward Euler method for both the normal and tangential velocity equations. The second scheme, which we denote by CNBE, uses a Crank-Nicolson scheme for the normal equations and a backward Euler method for the tangential equations. It may appear odd that we have exclusively used a first-order scheme for the tangential equations. However, as mentioned earlier, the tangential position of the mesh points should not have a major effect on the overall shape of the curve because this is determined by its normal velocity. The backward Euler scheme has therefore been chosen because it has better stability properties compared to the Crank--Nicolson scheme. Numerical experiments in the next section indicate that the CNBE scheme is second-order accurate in terms of the enclosed area error measure.

The curvature-based monitor function (2.19) requires an approximation of curvature. Based on a central difference approximation of (2.4) we set

(3.6) $$\kappa_{i}^{n}=\frac{4\left({\text{x}}_{i-1}^{n}-2{\text{x}}_{i}^{n}+{\text{x}}_{i+1}^{n}\right).{\text{n}}_{i}^{n}}{{\left|{\text{x}}_{i+1}^{n}-{\text{x}}_{i-1}^{n}\right|}^{2}}.$$

For the time-dependent floor (2.20) we use a simple quadrature approximation, and hence the discrete approximation of the monitor function (2.19) takes the form

(3.7) $$M_{i}^{n}=\frac{1}{2\left|\Gamma_{h}\left(t^{n}\right)\right|}\sum_{j=1}^{N}\left(\frac{{\left|\kappa_{j+1}^{n}\right|}^{1/2}+{\left|\kappa_{j}^{n}\right|}^{1/2}}{2}\right)h_{j}^{n}+\frac{1}{2}{\left|\kappa_{i}^{n}\right|}^{1/2},$$

where

(3.8) $$\left|\Gamma_{h}\left(t^{n}\right)\right|=\sum_{j=1}^{N}h_{j}^{n}.$$

To enhance the robustness of the adaptive grid procedure, we smooth the monitor function by using a spatial averaging technique [7], so that

(3.9) $${\tilde{M}}_{i}=\frac{\sum_{k=i-p}^{i+p}M_{k}{\left(q/\left(q+1\right)\right)}^{\left|k-i\right|}}{\sum_{k=i-p}^{i+p}{\left(q/\left(q+1\right)\right)}^{\left|k-i\right|}},$$

where *q* is a positive real number and *p* is a nonnegative integer. For the simulations presented later we set *p* = 2 and *q* = 3.

### 3.1 Initial grid generation

To initiate the moving mesh method, it is important to be able to generate a starting mesh which equidistributes the monitor function. This ensures a smooth initial evolution of the mesh points and improves solution accuracy and stability. We will assume that the initial curve can be expressed in terms of the parameterized variable *u*. Of course a uniform partition of the *u* domain is unlikely to equidistribute the given monitor function. We therefore need to generate a partition ${\left\{u_{i}\right\}}_{i=0}^{N}$ such that

$$\int_{0}^{u_{i}}M\left(u\right)\left|{\text{x}}_{u}\right|\text{d}u=\frac{i}{N}\int_{0}^{1}M\left(u\right)\left|{\text{x}}_{u}\right|\text{d}u,\;i=0,\ldots ,N.$$

To generate an approximation of the equdistributing mesh we will use an adaptation of the so-called de Boor algorithm [10]. We assume that *M* and |***x**_u_*| can be evaluated on an arbitrary background partition ${\left\{u_{i}^{\text{old}}\right\}}_{i=0}^{N}$ and that the function *M* (*u*)*|**x**_u_|* is approximated by the piecewise constant function

$$\rho \left(u\right)=\{\begin{array}{cc} M\left(u_{1/2}\right){\left|{\text{x}}_{u}\right|}_{1/2}, & u\in \left[u_{0},u_{1}\right],\\ M\left(u_{3/2}\right){\left|{\text{x}}_{u}\right|}_{3/2}, & u\in \left[u_{1},u_{2}\right],\\ \vdots & \vdots \\ M\left(u_{N-1/2}\right){\left|{\text{x}}_{u}\right|}_{N-1/2}, & u\in \left[u_{N-1},u_{N}\right], \end{array}$$

where *u*_*i*+1/2_ = (*u_i_* + *u*_*i*+1_)/2, *i* = 0, …, *N* − 1. A new partition ${\left\{u_{i}^{\text{new}}\right\}}_{i=0}^{N},$ which exactly equidistributes *ρ*(*u*), can be found using inverse linear interpolation (algorithmic details can be found in [31, 19]). The new partition of course only equidistributes *ρ* over the old partition, and hence iteration is used to update the partition further by simply setting the old partition to be the new partition and repeating the de Boor step. We can then generate a sequence of partitions that eventually converges to the final approximately equidistributed partition of the parameterized domain. The physical mesh point locations ${\left\{x\left(u_{i}\right),y\left(u_{i}\right)\right\}}_{i=0}^{N}$ are obtained from the parametric map of the final converged partition ${\left\{u_{i}\right\}}_{i=0}^{N}.$

## 4 Numerical experiments

### 4.1 Curve shortening flow of a circle

We first consider an initial unit circle shrinking according to curve shortening flow $\dot{\text{x}}.\text{n}=\kappa .$ The exact solution of this problem is a circle of radius $r\left(t\right)=\sqrt{1-2t,}$ and simulations were performed up to time *T* = 0.25. We define the error in the approximation of the enclosed area at time *t* ∈ (0, *T*] by *e_h_*(*t*) := *A_h_*(*t*) − *A*(*t*), where *A*(*t*) = *A*(0) − 2*πt* is the exact enclosed area for any closed curve evolved by classical curve shortening flow (and therefore will be used for other examples), and *A_h_* is the enclosed area for a polygon

$$A_{h}\left(t\right)=\frac{1}{2}\sum_{i=0}^{N-1}\left(x_{i}\left(t\right)y_{i+1}(t)-x_{i+1}\left(t\right)y_{i}\left(t\right)\right).$$

Convergence will be studied using the *L*^2^([0, *T*]) norm

$${\left\|e_{h}\right\|}_{L^{2}}\approx \sqrt{\sum_{n=1}^{N_{\text{T}}}{\left(A_{h}\left(t^{n}\right)-A\left(t^{n}\right)\right)}^{2}}\Delta t.$$

**Fig. 2:**
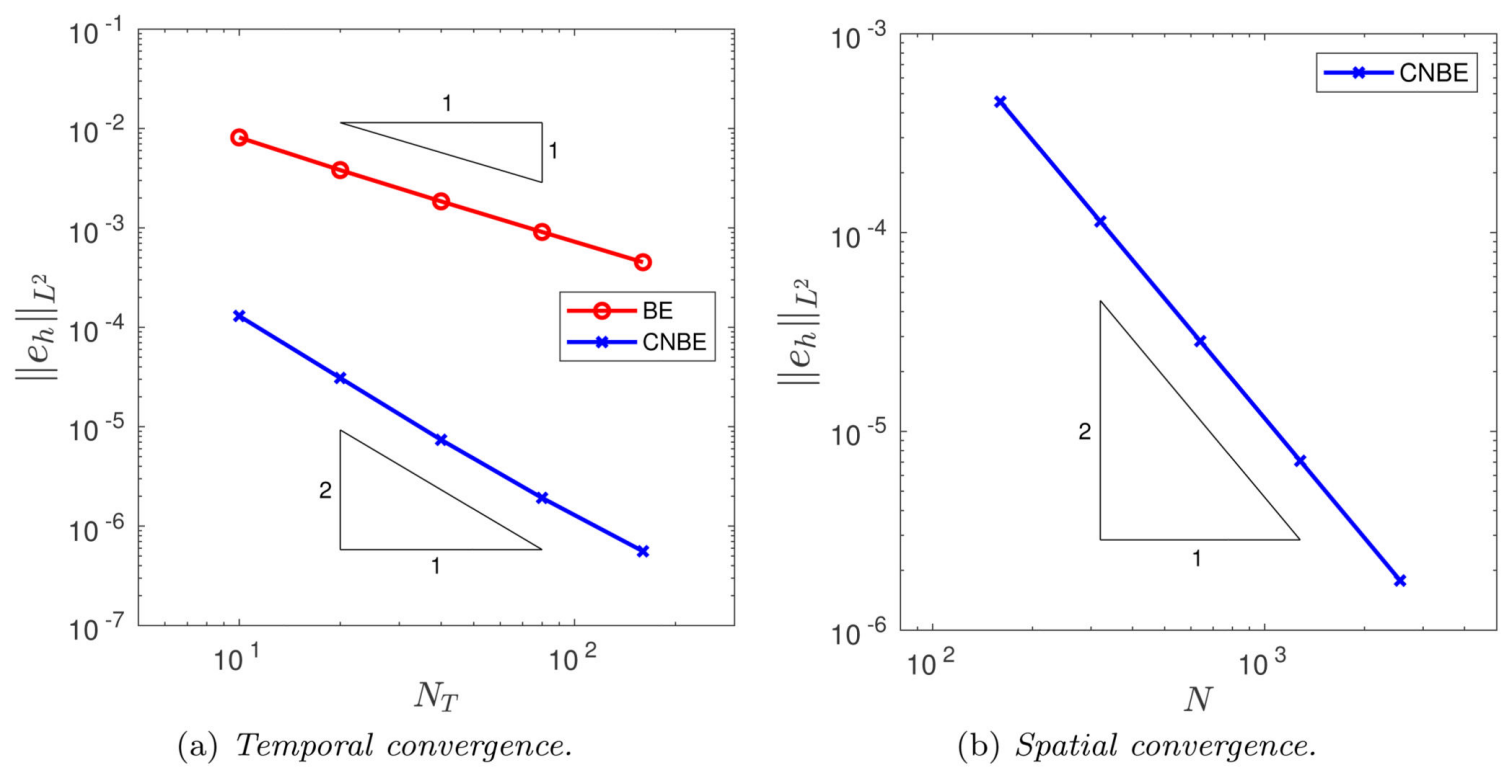
(a) Temporal and (b) spatial convergence in the *L*^2^ norm of the approximation of the enclosed area when an initial circle is evolved by curve shortening flow.

To test the temporal rate of convergence of the two time integration schemes, backward Euler (BE) and Crank–Nicolson backward Euler (CNBE), simulations were performed using a fine spatial mesh with *N* = 10^4^ points. The de Boor algorithm was used to construct the initial mesh, and we found no difference between the convergence properties of the two time integration schemes for any choice of spatial balancing operator *P* nor any choice of mesh relaxation time *τ*. Additionally, we found no difference in the convergence for each choice of monitor function *M*. Therefore, for the convergence studies we restrict ourselves (for brevity) to *M* = 1, *P* = 1, and *τ* = 0.1. Figure 2(a) shows the decrease in the error as the number of time steps is increased. As expected, the BE scheme is first-order convergent, while the CNBE scheme is second-order convergent. Furthermore, the CNBE scheme is considerably more accurate than the BE scheme using the equivalent number of time steps. These results highlight the improvement in accuracy achievable using a higher-order time integration scheme for the normal velocity equations. Spatial convergence was tested for the CNBE scheme using a large number of time steps *N_T_* = 10^4^ (i.e., Δ*t* = 2.5 × 10^−5^). As shown in Figure 2(b), the rate of spatial convergence is second order.

Each of the temporal integration schemes proposed requires the solution of a nonlinear system to evolve the solution forward in time. Table 1 displays the maximum and minimum number of Picard iterations required for each scheme, with *N* = 10^4^, for each temporal resolution considered. Clearly, the computational cost of solving the nonlinear system each time step is small. The maximum number of Picard iterations for the BE scheme is always greater than (or equal to) the maximum for the CNBE scheme. Note that for both the BE scheme and the CNBE scheme, the maximum number of Picard iterations decreases as *N_T_* is increased. Table 2 displays the maximum and minimum number of Picard iterations required for each scheme, with *N_T_* = 10^4^, for each spatial resolution. Once again, it is clear that the computational cost of solving the nonlinear system is minimal, requiring only two iterations per time step.

For this problem, there is no tangential movement of grid nodes because they move entirely in the normal direction to maintain a uniform arc-length distribution between mesh points. Additionally, due to the constant curvature in this example, there is no tangential movement of the grid nodes for the curvature-based monitor function. Therefore, we next consider an example with nonconstant curvature to assess the impact of the curvature-based monitor function on the accuracy of the two time integration schemes.

### 4.2 Curve shortening flow from an ellipse

We next consider curve shortening flow of an ellipse described parametrically by

(4.1) $$\begin{array}{l} x\left(u,0\right)=3\cos\left(2\pi u\right),\;0\leq u\leq 1,\\ y\left(u,0\right)=\sin\left(2\pi u\right). \end{array}$$

**Fig. 3:**
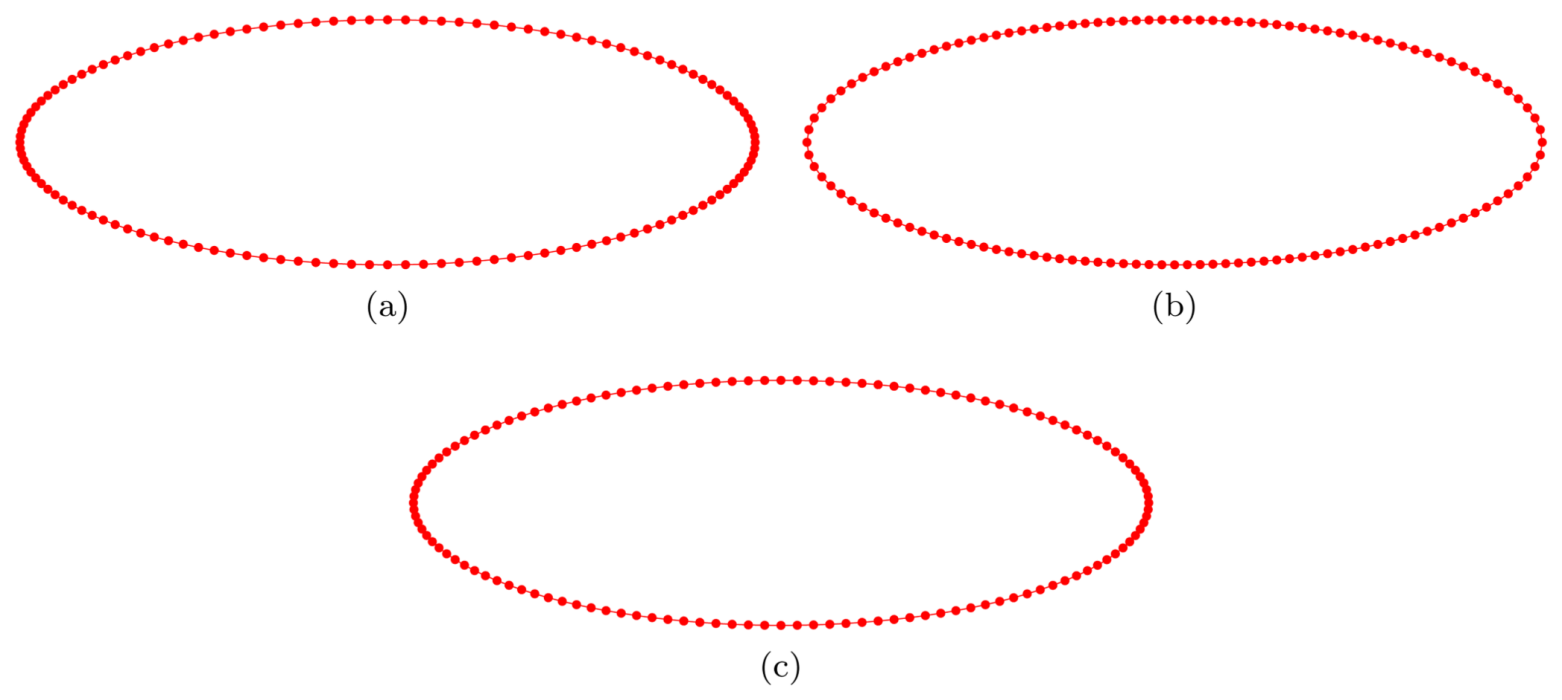
Initial mesh partitioning of the ellipse (4.1), using *N* = 128 points, according to (a) a uniform u-parameterization, (b) an equidistributed uniform arc-length approximation *M* = 1, and (c) an equidistributed curvature-based monitor function $M=\frac{1}{2}\left(M_{\text{floor}}+|\kappa {|}^{1/2}\right).$

Figure 3 illustrates the initial mesh partitioning of the ellipse (4.1), using *N* = 128 points, according to a uniform *u*-parameterization (Figure 3(a)), an equidistributed uniform arc-length approximation (Figure 3(b)), and an equidistributed curvature-based monitor function $M=\frac{1}{2}\left(M_{\text{floor}}+{\left|\kappa \right|}^{1/2}\right)$ (Figure 3(c)). Although grid points for the uniform *u*-parameterization and the equidistributed curvature-based monitor function may seem similar, the use of an initial mesh that equidistributes the given monitor function turns out to be important, which we will see later.

**Fig. 4:**
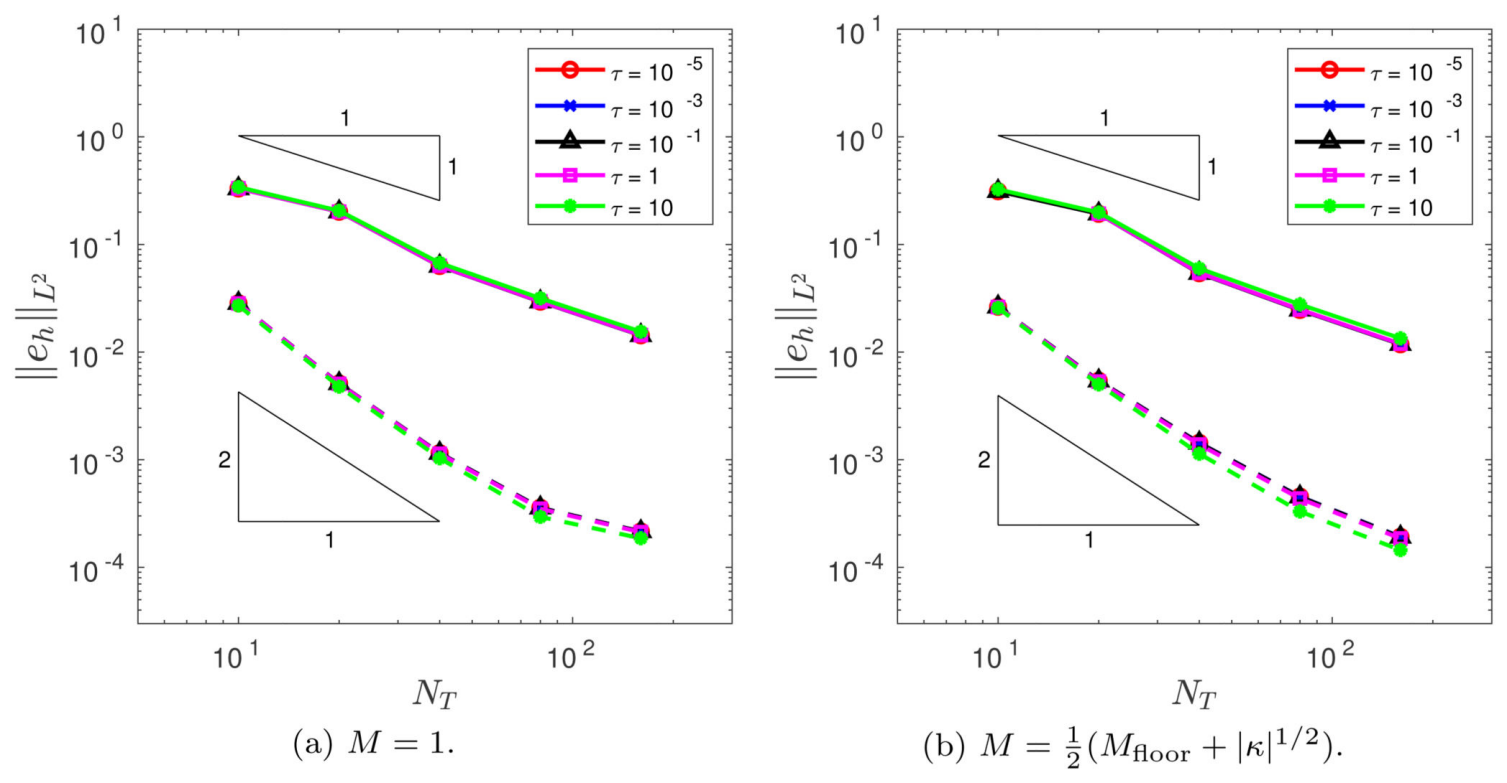
Temporal convergence in the *L*^2^([0, *T*]) norm of the approximation of the enclosed area when an initial ellipse is evolved by curve shortening flow using the BE (solid line) and the CNBE (dashed line) scheme with *P* = *M*|***x***_*ξ*_|^2^ and (a) *M* = 1 or (b) $M=\frac{1}{2}\left(M_{\text{floor}}+|\kappa {|}^{1/2}\right).$

The initial position of the grid nodes is determined by the de Boor algorithm (section 3.1). In all simulations, we run to a final time of *T* = 1.4. Throughout this section, we assume a spatial balancing operator of the form *P* = *M*|***x**_ξ_*|^2^. The temporal convergence was tested for both the BE and CNBE schemes, using a fine mesh resolution of *N* = 10^3^. Figure 4(a) illustrates the temporal convergence when *M* = 1. It is clear that the BE scheme demonstrates first-order convergence and the CNBE scheme demonstrates second-order convergence. (The slight flattening out of the error decrease for large values of *N_T_* is due to the pollution of the global error by spatial error components.) Figure 4(b) illustrates the temporal convergence when $M=\frac{1}{2}\left(M_{\text{floor}}+{\left|\kappa \right|}^{1/2}\right)$. Once again, it is clear that the BE scheme demonstrates first-order convergence and the CNBE scheme demonstrates second-order convergence. Additionally, Figures 4(a) and 4(b) demonstrate that, for our choice of spatial balancing operator *P* = *M*|***x**_ξ_*|^2^, the errors are robust to the choice of *τ*.

Following the circle example (section 4.1), we wish to illustrate the computational efficiency of the nonlinear Picard solver. Thus, for both monitor functions, Table 3 displays the maximum and minimum number of Picard iterations required for each scheme and for each temporal resolution considered when *τ* = 10. Apart from when *N_T_* = 10 (which corresponds to the largest time step size), the computational cost of the CNBE scheme is reasonably low. Additionally, we note that using a curvature-based monitor function does not substantially increase the computational cost of the Picard solver. For *N_T_* = 10, 20 we note that the BE scheme struggles compared with the CNBE scheme. Indeed, for the BE scheme, when *N_T_* = 20 and the curvature-based monitor function is used, there is an increase in the maximum number of Picard iterations. This spike is reflected in the convergence plots (Figure 4), where there is a slight bump for *N_T_* = 20. However, as the number of time steps increases, the maximum number of Picard iterations decreases for both schemes.

**Fig. 5:**
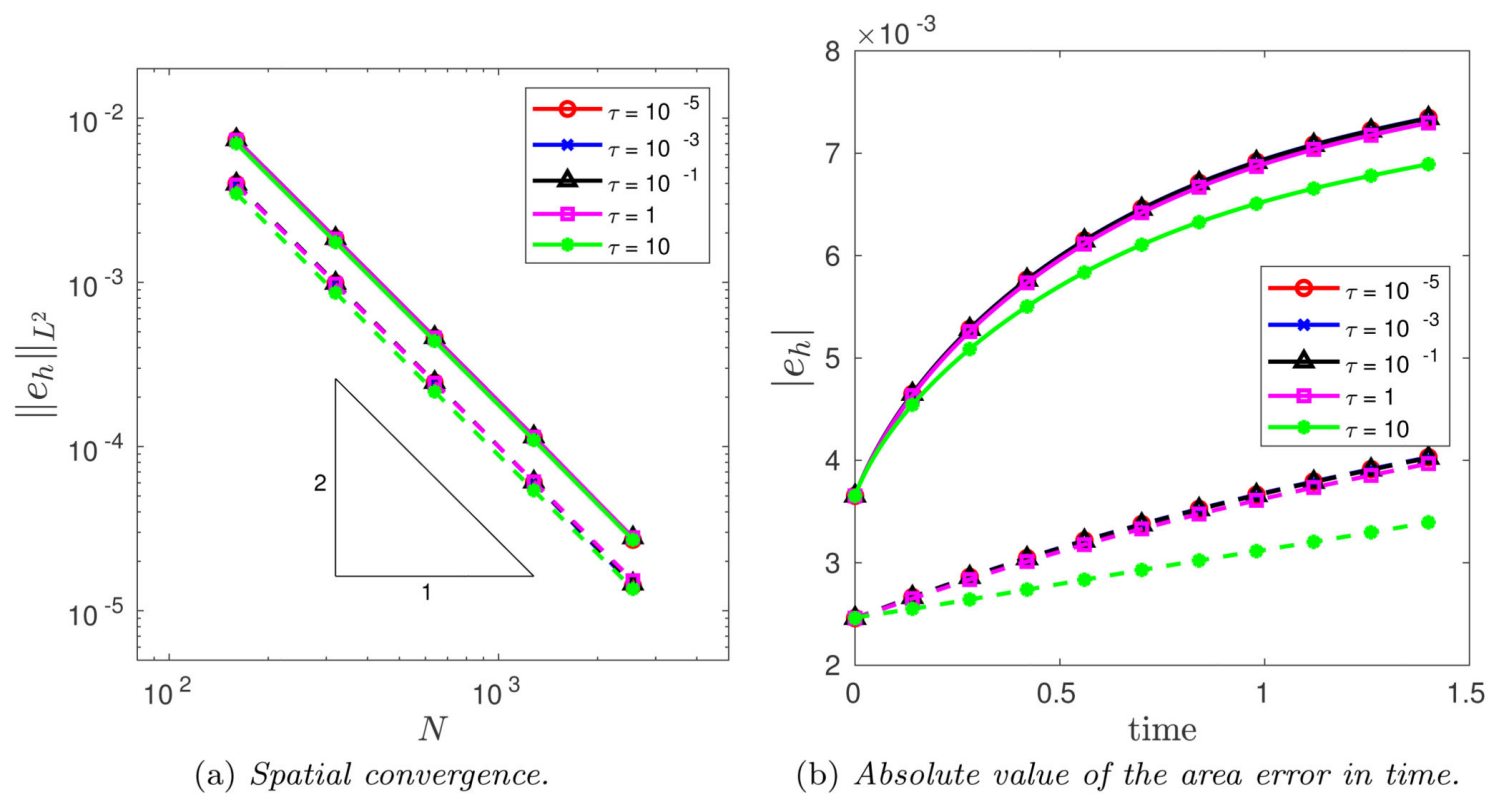
(a) Spatial convergence in the *L*^2^([0, *T*]) norm of the approximation of the enclosed area when an initial ellipse is evolved by curve shortening flow using the CNBE scheme for all *τ* and for both *M* = 1 (solid line) and $M=\frac{1}{2}\left(M_{\text{floor}}+|\kappa {|}^{1/2}\right)$ (dashed line). (b) Absolute value of the area error in time for the CNBE scheme with *N* = 160 for both *M* = 1 (solid line) and $M=\frac{1}{2}\left(M_{\text{floor}}+|\kappa {|}^{1/2}\right)$ (dashed line).

The spatial convergence was tested using a large number of time steps *N_T_* = 10^4^. Figure 5(a) illustrates the spatial convergence of the CNBE scheme when *M* = 1 (solid line) and $M=\frac{1}{2}\left(M_{\text{floor}}+{\left|\kappa \right|}^{1/2}\right)$ (dashed line). The convergence is clearly second order for all values of *τ* for both *M* = 1 and $M=\frac{1}{2}\left(M_{\text{floor}}+{\left|\kappa \right|}^{1/2}\right)$ but crucially, the curvature-based monitor function produces an improved error compared with the uniform arc-length monitor function. This is due to the curve being more accurately approximated using a curvature-based monitor function compared with uniform arc-length. To this end, Figure 5(b) illustrates the absolute value error in the computed area for the same spatial balancing operator for both *M* = 1 and $M=\frac{1}{2}\left(M_{\text{floor}}+{\left|\kappa \right|}^{1/2}\right)$. Once again, it is clear that the curvature-based monitor function produces a much better mesh compared to a uniform arc-length.

As was demonstrated in the temporal convergence study, Figure 5(a) shows that, for a given monitor function, all values of *τ* perform similarly. Thus, for both monitor functions, Table 4 displays the maximum and minimum number of Picard iterations required for each scheme and for each spatial resolution considered when *τ* = 10. As was seen for the circle example (section 4.1), the computational cost of the nonlinear Picard solver is minimal, requiring at most three iterations, and increasing the spatial resolution does not affect the maximum number of iterations.

#### 4.2.1 Uniform *u*-parameterization

In the previous section, we used the de Boor algorithm to construct the initial approximation to a given curve. An obvious question is, Why is this necessary? Therefore, in this section we provide evidence for the importance of using an equidistributed initial grid. Indeed, here the initial position of the grid nodes is given by a uniform *u*-parameterization described by (4.1) and illustrated in Figure 3(a). It is clear from Figure 3(a) that the initial distribution of the grid nodes resulting from a uniform *u*-parameterization is similar to the distribution obtained from an equidistributed curvature-based monitor function (Figure 3(c)). In general, this will not be the case (as will be shown later). Therefore, to emphasize the importance of using an equidistributed initial grid we will restrict ourselves to the uniform arc-length monitor function *M* = 1 (Figure 3(b)).

**Fig. 6:**
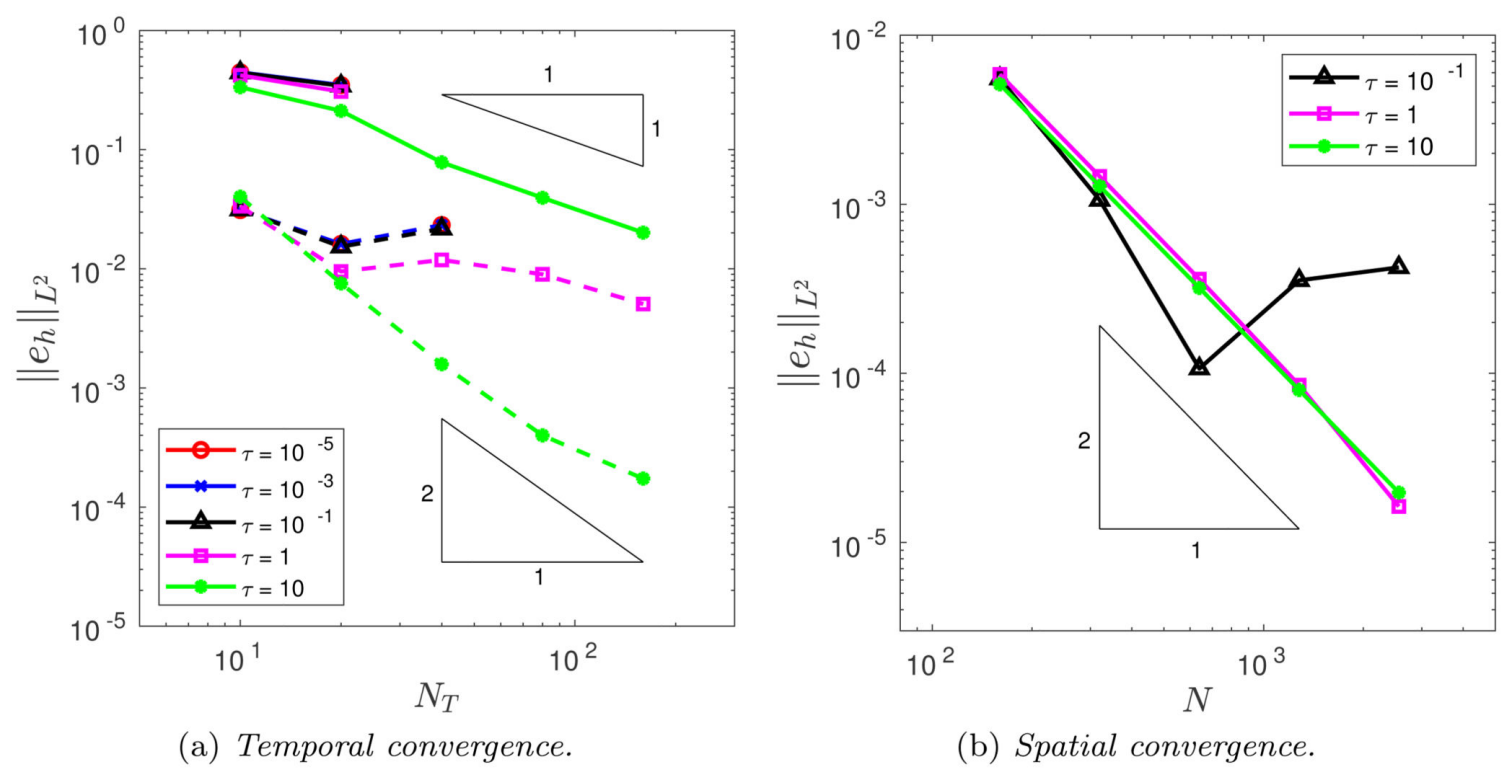
(a) Temporal and (b) spatial convergence in the *L*^2^([0, *T*]) norm of the approximation of the enclosed area when an initial ellipse is evolved by curve shortening flow with *M* = 1 and *P* = *M*|***x***_ξ_|^2^.

Figure 6(a) illustrates the temporal convergence when *M* = 1 and *P* = *M*|***x**_ξ_*|^2^ for both the BE (solid line) and CNBE (dashed line) schemes. From Figure 6(a), it is clear that the only value of *τ* to obtain the expected convergence for either the BE or CNBE scheme is *τ* = 10. For the CNBE scheme, when *τ* = 1 we see pre-asymptotic convergence, while no convergence is possible for the other values of *τ* because the nonlinear Picard solver is unable to converge within the maximum number of iterations. The BE scheme cannot obtain convergence for any value of *τ* except *τ* = 10. Evidently the choice of *τ* is important for the time scales considered. Taking too small a value of *τ* in relation to Δ*t* pollutes the convergence rate significantly and also increases the computational cost of the Picard solver, to the point where the solver may not converge at all. Although the precise relationship between *τ* and Δ*t* is not currently known, we hypothesize that the ratio between *τ* and Δ*t* is of greater importance than the individual values, and generally one should avoid having *τ ≪* Δ*t*.

**Fig. 7:**
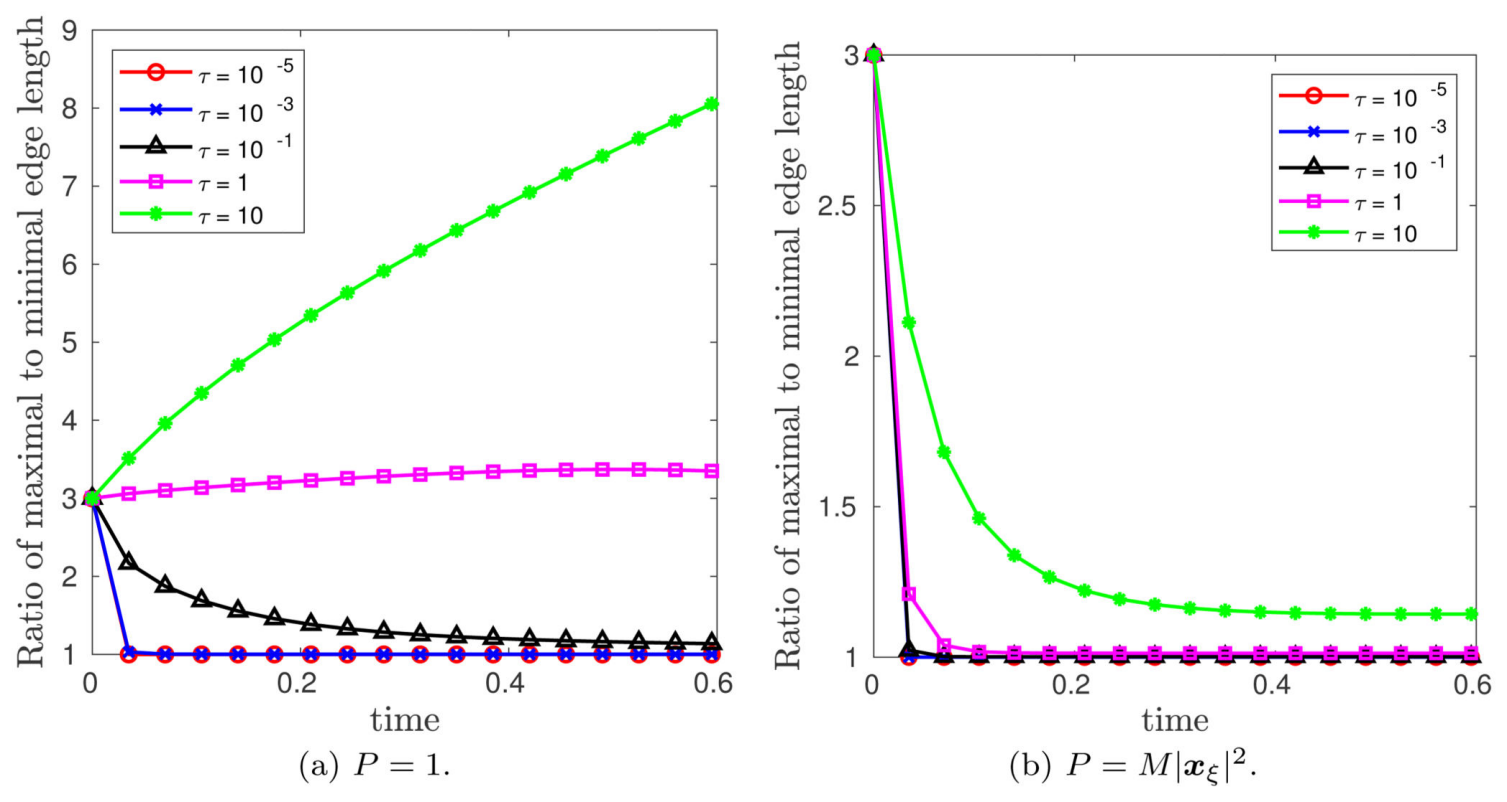
(Ellipse.) Ratio of maximal to minimal edge length in time when *M* = 1, *N* = 10^3^, *N_T_* = 40 with (a) *P* = 1 and (b) *P* = *M*|***x***_*ξ*_|^2^.

Figure 6(b) illustrates the spatial convergence when *M* = 1 and *P* = *M*|***x**_*ξ*_*|^2^ for the CNBE scheme. It is clear that *τ* = 1 and *τ* = 10 achieve the expected second-order convergence. However, no convergence was possible for *τ* = 10^−5^ and *τ* = 10^−3^, and pre-asymptotic convergence was seen for *τ* = 0.1. To illustrate the role of the mesh relaxation time *τ*, Figure 7 plots the ratio of the maximal to minimal edge length of the evolving mesh in time. First, when *P* = 1, Figure 7(a) demonstrates that when *τ* = 10^−5^ or *τ* = 10^−3^ the MMPDE equidistributes arc-length within a few time steps. At first glance, a quick convergence to a uniform arc-length mesh appears desirable, but this rapid equidistribution results in a loss of smoothness in the nodal trajectories. Therefore, having an initially well-defined grid, constructed by equidistributing a given monitor function, will help prevent rapid equidistribution of the nodal points in time and thus increase the robustness of the algorithm by reducing the dependency on the mesh relaxation time *τ*. Also illustrated in Figure 7(a) is the potential negative effect of having too large a value of *τ*. Too large a value could prevent the MMPDE from equidistributing a given monitor function in time and hence produce undesirable nodal clustering.

From Figure 6, when *P* = *M*|***x**_*ξ*_*|^2^ we expect to see a rapid equidistribution within a few time steps for *τ* ≤ 1, and this is precisely what is seen in Figure 7(b). These results suggest that the spatial balancing operator *P* = *M*|***x**_*ξ*_*|^2^ alters the time scale of mesh relaxation, allowing larger values of *τ* to be used. Incidentally, this explains why the spatial balancing operator *P* = *M*|***x**_*ξ*_*|^2^ was chosen here. As just discussed, having an initially equidistributed grid prevents rapid equidistribution of the nodal points in time and thus increases the robustness of the algorithm by allowing smaller values of *τ*, while the spatial balancing operator *P* = *M*|***x**_*ξ*_*|^2^ allows larger values of *τ* as illustrated in Figures 6 and 7(b). These two observations combined enable us to minimize the dependency on the mesh relaxation time *τ*. This is illustrated in the temporal (Figure 4) and spatial (Figure 5(a)) convergence plots given previously, where each value of *τ* performed similarly.

### 4.3 Curve shortening flow of a nonconvex initial curve

We next consider curve shortening flow of the nonconvex initial curve

(4.2) $$\begin{array}{l} x(u,0)=\cos(2\pi u),\;0\leq u\leq 1,\\ y(u,0)=0.5\;\text{sin}(2\pi u)+\text{sin}(\text{cos}(2\pi u))+\text{sin}(2\pi u)(0.2+\text{sin}(2\pi u){\;\text{sin}}^{2}(6\pi u)). \end{array}$$

This example was used previously to test tangentially stabilized curve evolution algorithms [2, 3, 24, 33].

**Fig. 8:**
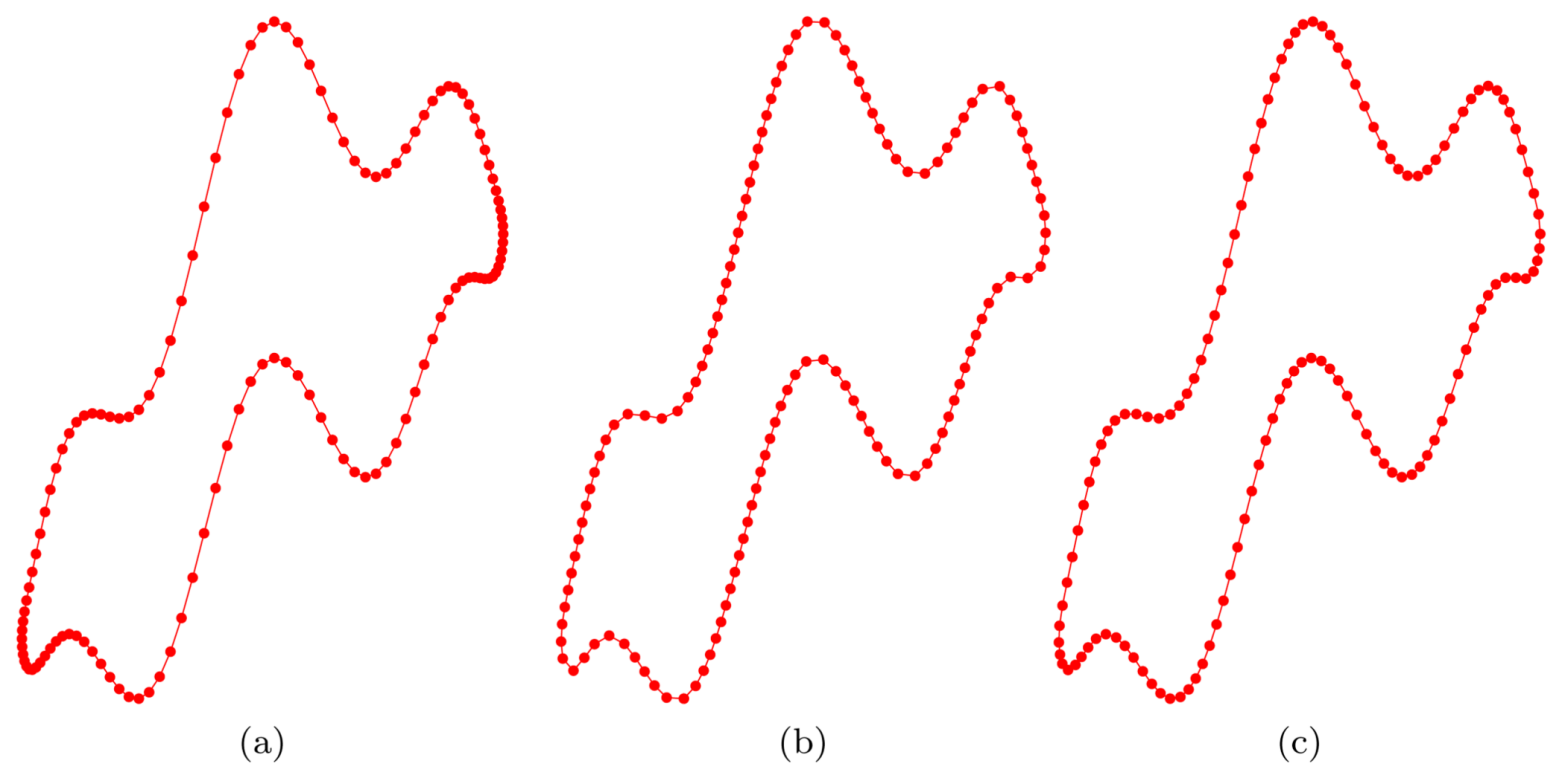
Initial mesh partitioning of the nonconvex curve (4.2), using *N* = 128 points, according to (a) a uniform u-parameterization, (b) an equidistributed uniform arc-length approximation *M* = 1, and (c) an equidistributed curvature-based monitor function $M=\frac{1}{2}\left(M_{\text{floor}}+|\kappa {|}^{1/2}\right).$

Figure 8 illustrates the initial mesh partitioning of the nonconvex curve (4.2), using *N* = 128 points. From Figure 8(a), we can see that when the initial mesh is obtained using a uniform *u*-parameterization, the distribution of points is far from ideal, with some areas of high curvature having poor resolution while others have severe nodal clustering. Similarly, Figure 8(b) illustrates that an equidistributed uniform arc-length mesh is also poor at resolving areas of high curvature. The best initial mesh is obtained from an equidistributed curvature-based monitor function $M=\frac{1}{2}\left(M_{\text{floor}}+{\left|\kappa \right|}^{1/2}\right)$ (Figure 8(c)), where we can observe a good balance of the distribution of mesh points towards high-curvature regions and areas of low curvature.

**Fig. 9:**
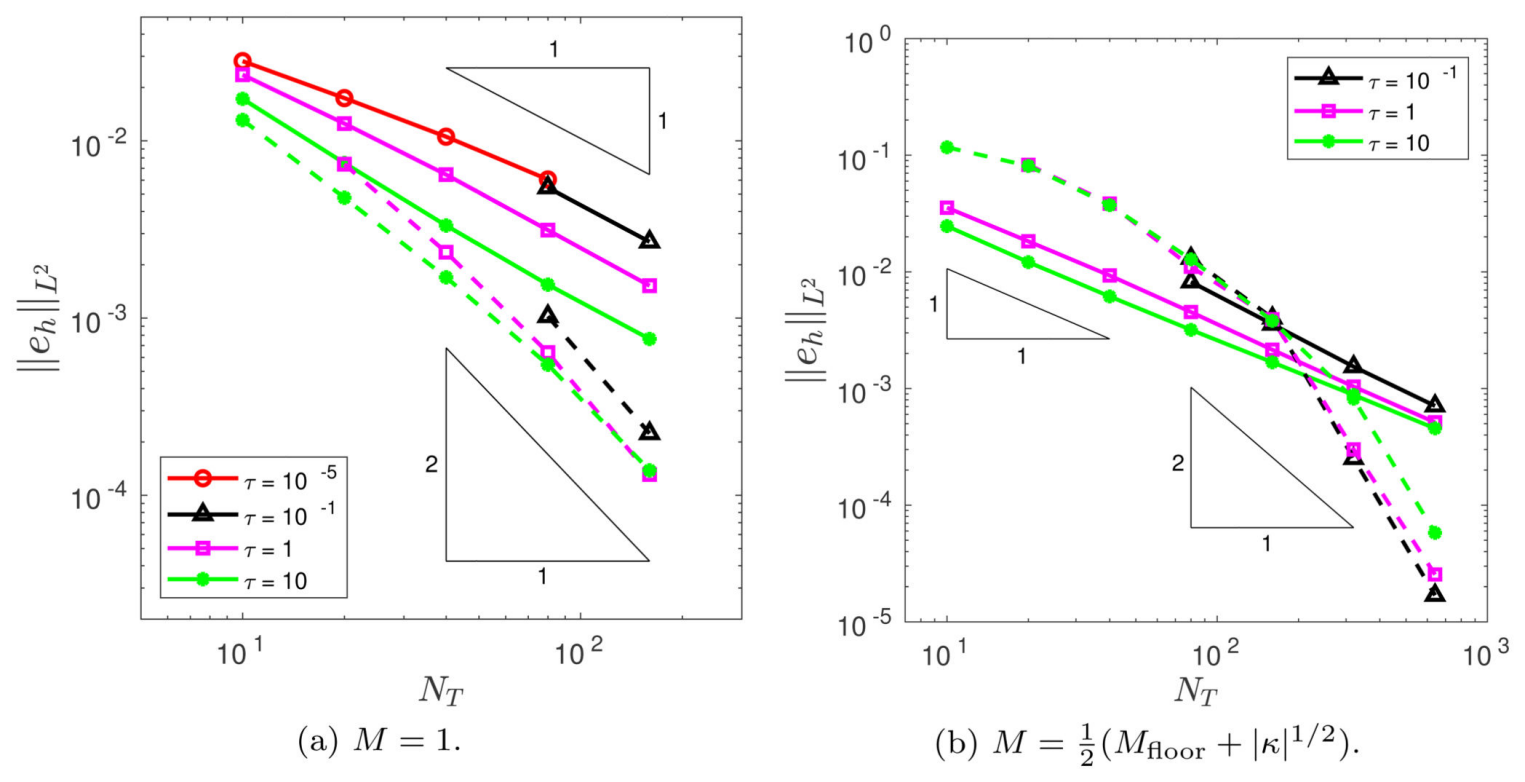
Temporal convergence in the *L*^2^([0, *T*]) norm of the approximation of the enclosed area when the nonconvex initial curve is evolved by curve shortening flow using the BE (solid line) and the CNBE (dashed line) scheme with *P* = *M*|***x***_*ξ*_|^2^ and (a) *M* = 1 or (b) $M=\frac{1}{2}\left(M_{\text{floor}}+|\kappa {|}^{1/2}\right).$

Following the ellipse example (section 4.2), the initial position of the grid nodes is determined by the de Boor algorithm (section 3.1). In all simulations, we run to a final time of *T* = 0.25. Once again, we assume a spatial balancing operator of the form *P* = *M*|***x**_*ξ*_*|^2^. The temporal convergence was tested for both the BE and CNBE schemes, using a fine mesh resolution of *N* = 10^3^. Figure 9(a) illustrates the temporal convergence when *M* = 1. It is clear that the BE scheme demonstrates first-order convergence for *τ* = 1 and *τ* = 10 while giving only partial results for *τ* = 10^−5^ and *τ* = 0.1. No convergence results were obtained for *τ* = 10^−3^. For *τ* = 1, the CNBE scheme demonstrates approximate second-order convergence and slightly less than second-order convergence for *τ* = 10. Only partial results were obtained for *τ* = 0.1, and no convergence is seen for *τ* = 10^−5^ and *τ* = 10^−3^. Figure 9(b) illustrates the temporal convergence when $M=\frac{1}{2}\left(M_{\text{floor}}+{\left|\kappa \right|}^{1/2}\right)$. Once again, it is clear that the BE scheme demonstrates first-order convergence for *τ* = 1 and *τ* = 10 but only partial results for *τ* = 0.1. Unlike when *M* = 1, convergence results could not be obtained for either *τ* = 10^−5^ or *τ* = 10^−3^. However, the CNBE scheme does not behave as we expect; the error for the CNBE is larger than that for the BE scheme for the smallest values of *N_T_*. This error then plummets, achieving greater than second-order accuracy. In this example, there is substantial tangential motion as the regions of very high curvature flatten out, and this may be a source of additional error compared to other examples.

Following the previous examples (sections 4.1 and 4.2), we wish to illustrate the computational efficiency of the nonlinear Picard solver. As can be seen from Figure 9, for each monitor function, all values of *τ* do not perform similarly enough, with respect to the *L*^2^ error measure, unlike the ellipse example (this is particularly true for the BE scheme). Therefore, for both monitor functions, we choose *τ* = 10 as this seems to perform more robustly. Tables 5 and 6 display the maximum and minimum number of Picard iterations required for each scheme and for each temporal resolution considered when *τ* = 10. For the smallest values of *N_T_* (Table 5), we see that both the BE and CNBE schemes slightly struggle for both the uniform arc-length and curvature-based monitor functions. However, we note that as *N_T_* is increased, the maximum number of Picard iterations decreases. It is therefore not surprising that the computational cost for the nonlinear Picard solver becomes far more reasonable for the largest values of *N_T_* (Table 6).

**Fig. 10:**
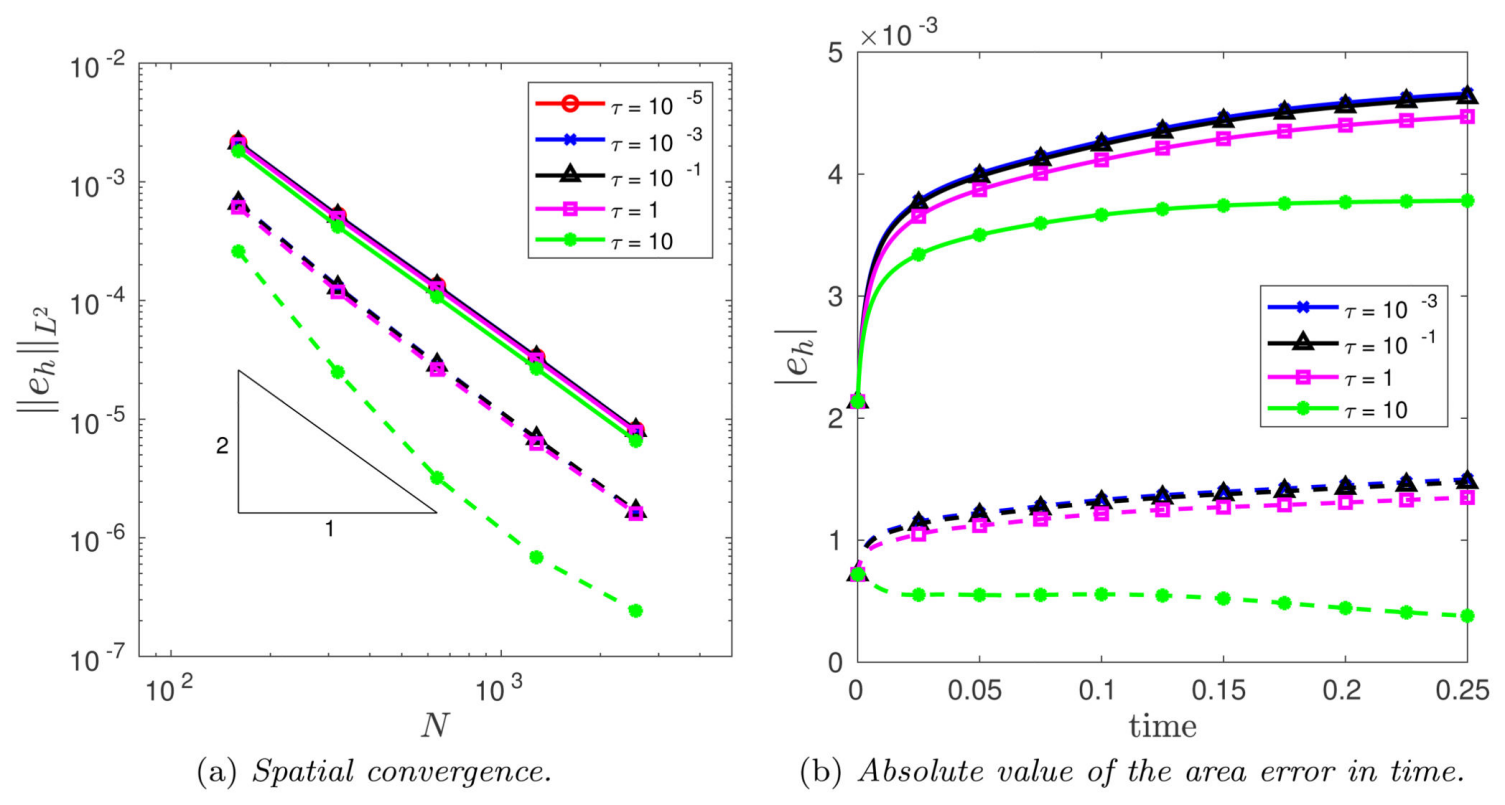
(a) Spatial convergence in the *L*^2^([0, *T*]) norm of the approximation of the enclosed area when the initial nonconvex curve is evolved by curve shortening flow using the CNBE scheme for all *τ* and for both *M* = 1 (solid line) and $M=\frac{1}{2}\left(M_{\text{floor}}+|\kappa {|}^{1/2}\right)$ (dashed line). (b) Absolute value of the area error in time for the CNBE scheme with *N* = 160 for both *M* = 1 (solid line) and $M=\frac{1}{2}\left(M_{\text{floor}}+|\kappa {|}^{1/2}\right)$ (dashed line).

The spatial convergence was once again tested using a large number of time steps *N_T_* = 10^4^. Figure 10(a) illustrates the spatial convergence of the CNBE scheme when *M* = 1 (solid line) and $M=\frac{1}{2}\left(M_{\text{floor}}+{\left|\kappa \right|}^{1/2}\right)$ (dashed line). The convergence is clearly second order for all values of *τ* for *M* = 1, while for $M=\frac{1}{2}\left(M_{\text{floor}}+{\left|\kappa \right|}^{1/2}\right)$ second-order convergence is seen for all *τ* except *τ* = 10, where greater than second-order convergence is seen. Crucially, however, the curvature-based monitor function produces an improved error compared with the uniform arc-length monitor function. This is due to the curve being more accurately approximated using a curvature-based monitor function compared to uniform arc-length. To this end, Figure 10(b) illustrates the absolute value error in the computed area for the same spatial balancing operator for both *M* = 1 and $M=\frac{1}{2}\left(M_{\text{floor}}+{\left|\kappa \right|}^{1/2}\right)$. Once again, it is clear that the curvature-based monitor function produces a much better mesh compared to a uniform arc-length.

Figure 10(a) shows that, for a given monitor function, all values of *τ* perform similarly except for the curvature-based monitor function when *τ* = 10, where greater than second-order convergence was seen. Thus, for both monitor functions, Table 7 displays the maximum and minimum number of Picard iterations required for each scheme and for each spatial resolution considered when *τ* = 1. As was seen for the previous examples (sections 4.1 and 4.2), the computational cost of the nonlinear Picard solver is low, requiring at most seven iterations.

### 4.4 Curve shortening flow with a singularity in finite time

We now consider curve shortening flow for the initial curve given by the parameterization

(4.3) $$\begin{array}{l} x(u,0)=\text{cos}(4\pi u)\;\text{cos}(2\pi u),\;0\leq u\leq 1,\\ y(u,0)=\text{cos}(4\pi u)\;\sin(2\pi u). \end{array}$$

The initial curve is self-intersecting and develops a geometric singularity in a finite time. Once again, the initial position of the grid nodes is determined by the de Boor algorithm, and we assume a spatial balancing operator of the form *P* = *M*|***x**_*ξ*_*|^2^. The simulation was run until *T* = 0.086.

**Fig. 11:**
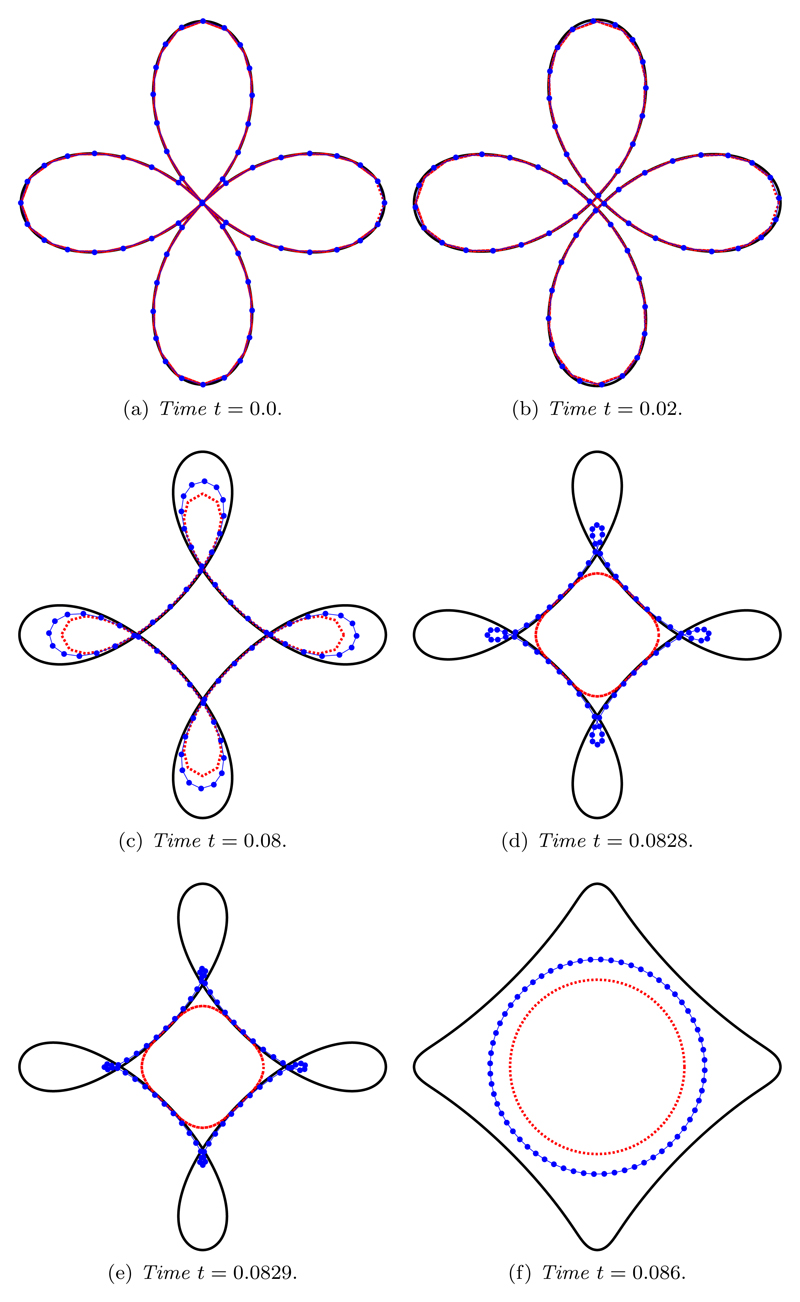
Curve shortening flow of the self-intersecting curve (4.3) using the CNBE scheme, for *τ* = 10^−3^ and Δ*t* = 10^−5^, comparing a gold standard approximation with *N* = 10^3^ (solid line) against a uniform arc-length approximation *M* = 1 (dotted line) and a curvature-based approximation $M=\frac{1}{2}\left(M_{\text{floor}}+|\kappa {|}^{1/2}\right)$ (line with markers). As in [15], images are rescaled. (See online version for color.)

Figure 11 illustrates a comparison of a *gold standard* approximation, with a fine spatial resolution *N* = 10^3^ (solid line), against a uniform arc-length approximation, where *M* = 1 (dotted line), and a curvature-based approximation, where $M=\frac{1}{2}\left(M_{\text{floor}}+{\left|\kappa \right|}^{1/2}\right)$ (line with markers). For the gold standard simulation we use *M* = 1 and note that, for a time step size of Δ*t* = 10^−5^, the simulation evolved smoothly for a choice of *τ* = 10^−3^. To allow comparison with the results presented in [15], the uniform arc-length and curvature-based simulations were carried out with *N* = 64. The time step size used in [15] is of the order Δ*t* = 10^−4^. To highlight the improvement from using a curvature-based grid over a uniform arc-length grid we use the same time step size as in the gold standard solution. The solutions are plotted at the same times as those in [15]. We can see that the coarse uniform arc-length grid is a reasonable approximation at *t* = 0.02, but due to the lack of resolution of high-curvature regions the accuracy deteriorates considerably to the extent that the singularity in the curve occurs between *t* = 0.08 (Figure 11(c)) and *t* = 0.0828 (Figure 11(d)). However, for the gold standard approximation, the singularity occurs between *t* = 0.0829 (Figure 11(e)) and *t* = 0.086 (Figure 11(f)). The curvature-based grid has an improved approximation when compared with the uniform arc-length grid for times *t* ≥ 0.08 due to the improved resolution of high-curvature regions afforded by a curvature-based grid. The singularity occurs between *t* = 0.0829 and *t* = 0.086. As demonstrated by Elliott and Fritz [15], it is not possible for an iterative, fully implicit scheme to converge past the singularity. In [15], a semi-implicit approach was used, which enabled the numerics to jump the geometric singularity. Here, the semi-implicit nature is achieved by restricting the number of iterations used in the nonlinear Picard solver. For the BE scheme, a semi-implicit approach can be obtained by allowing only a single Picard iteration, while for the CNBE scheme (depicted in Figure 11) this limit is two.

### 4.5 Forced curve shortening flow

Thus far we have only considered classical curve shortening flow $\dot{x}\cdot n=\kappa \text{.}$ In this section, we add a constant forcing so that

$$\dot{x}\cdot n=\alpha \kappa +\beta ,$$

where *α* = *α*(***x**, t*) = 1 and *β* = *β*(***x**, t*) = 10. Unfortunately, for forced curve shortening flow, we do not possess an exact solution for the evolving area. Therefore, we define the error in the approximation of the enclosed area at time *t* ∈ (0, *T*] by *e_h_*(*t*) := *A_h_*(*t*) − *A_gs_*(*t*), where *A_gs_*(*t*) is the enclosed area for the *gold standard* approximation and *A_h_* is as defined previously (see section 4.1). Convergence will be studied using the absolute value error

$$\left|e_{h}\right|=\left|A_{h}-A_{gs}\right|.$$

#### 4.5.1 Forced curve shortening flow of a unit *l_p_*-ball

In this section, our initial curve is defined as a unit *l_p_*-ball,

$$\Gamma_{p}=\left\{x\in {\mathbb{R}}^{2}:{\left\|x\right\|}_{p}=1\right\},$$

where for any ***x*** = (*x, y*) ∈ ℝ^2^, the *l_p_* norm is defined as ${\left\|x\right\|}_{p}={\left({\left|x\right|}^{p}+{\left|y\right|}^{p}\right)}^{1/p}\text{.}$ Following previous sections, we define the curve parametrically as

(4.4) $$\begin{array}{l} \begin{array}{ll} x\left(u,0\right)=r\left(u\right)\text{cos}\left(2\pi u\right), & 0\leq u\leq 1, \end{array}\\ \;y\left(u,0\right)=r\left(u\right)\text{sin}\left(2\pi u\right), \end{array}$$

where we assume that the radius depends on the parameter *u*. Substituting this into the definition of Γ_*p*_ yields the following expression for the radius:

(4.5) $$r(u)=\frac{1}{{\left(|\text{cos}(2\pi u){|}^{p}+|\text{sin}(2\pi u){|}^{p}\right)}^{1/p}}.$$

Throughout this section, we assume that *p* = 10.

**Fig. 12:**
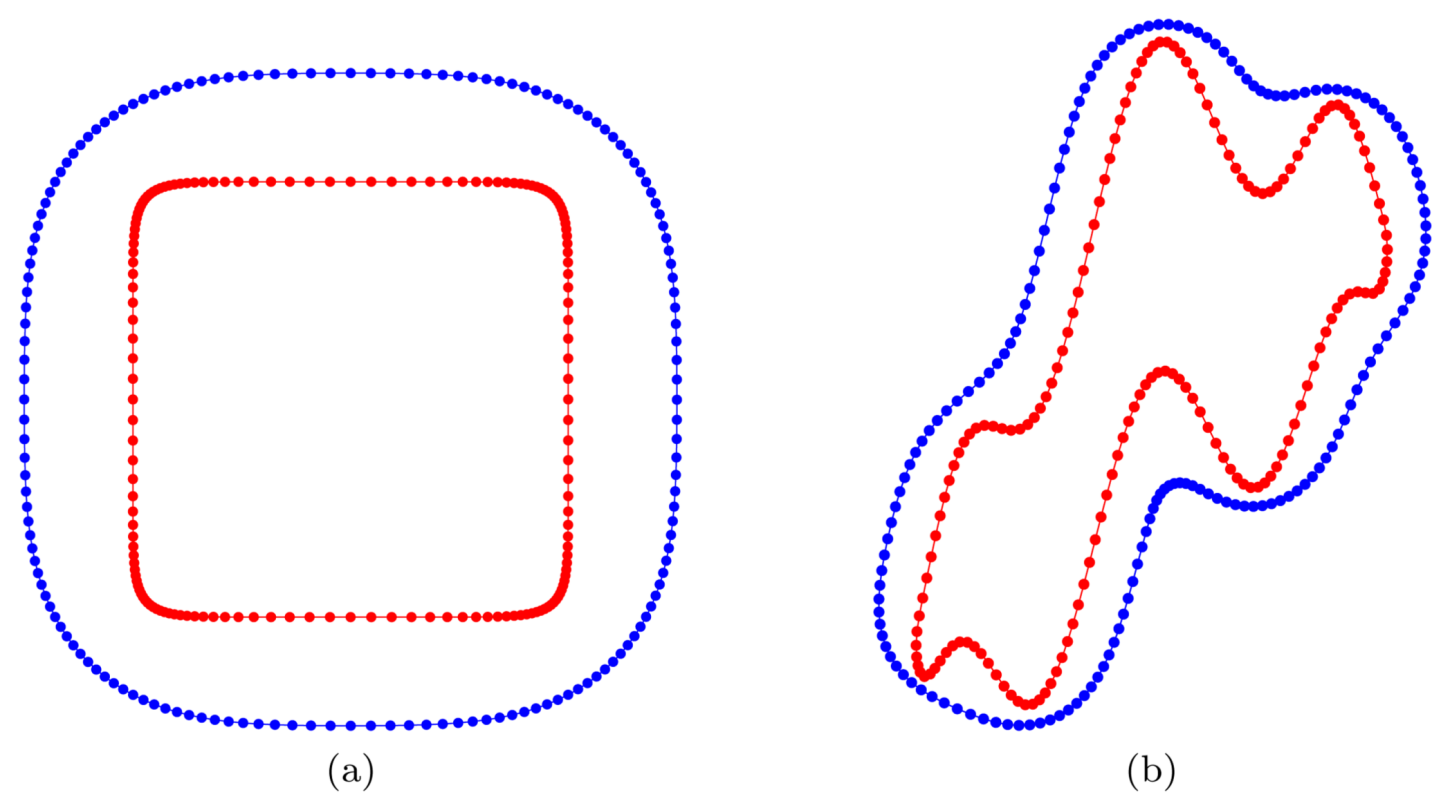
Initial (inner curve) and final (outer curve) mesh partitioning of (a) the *l_p_*-ball (4.4) and (b) the nonconvex initial curve (4.2) using *N* = 160 points. The initial mesh is obtained by equidistributing the curvature-based monitor function $M=\frac{1}{2}\left(M_{\text{floor}}+|\kappa {|}^{1/2}\right).$*)* (See online version for color.)

Figure 12(a) illustrates the initial (inner curve) and final (outer curve) mesh partitioning of the curve defined as an *l_p_*-ball of order *p* = 10 using *N* = 160 points. The initial mesh is obtained by equidistributing the curvature-based monitor function. Using the given values of the parameters *α* and *β*, it is clear that the addition of a constant forcing term causes the length of the curve to increase in time rather than decrease. Also, it is clear that the curvature-based monitor function obtains a high resolution around the high-curvature regions.

**Fig. 13:**
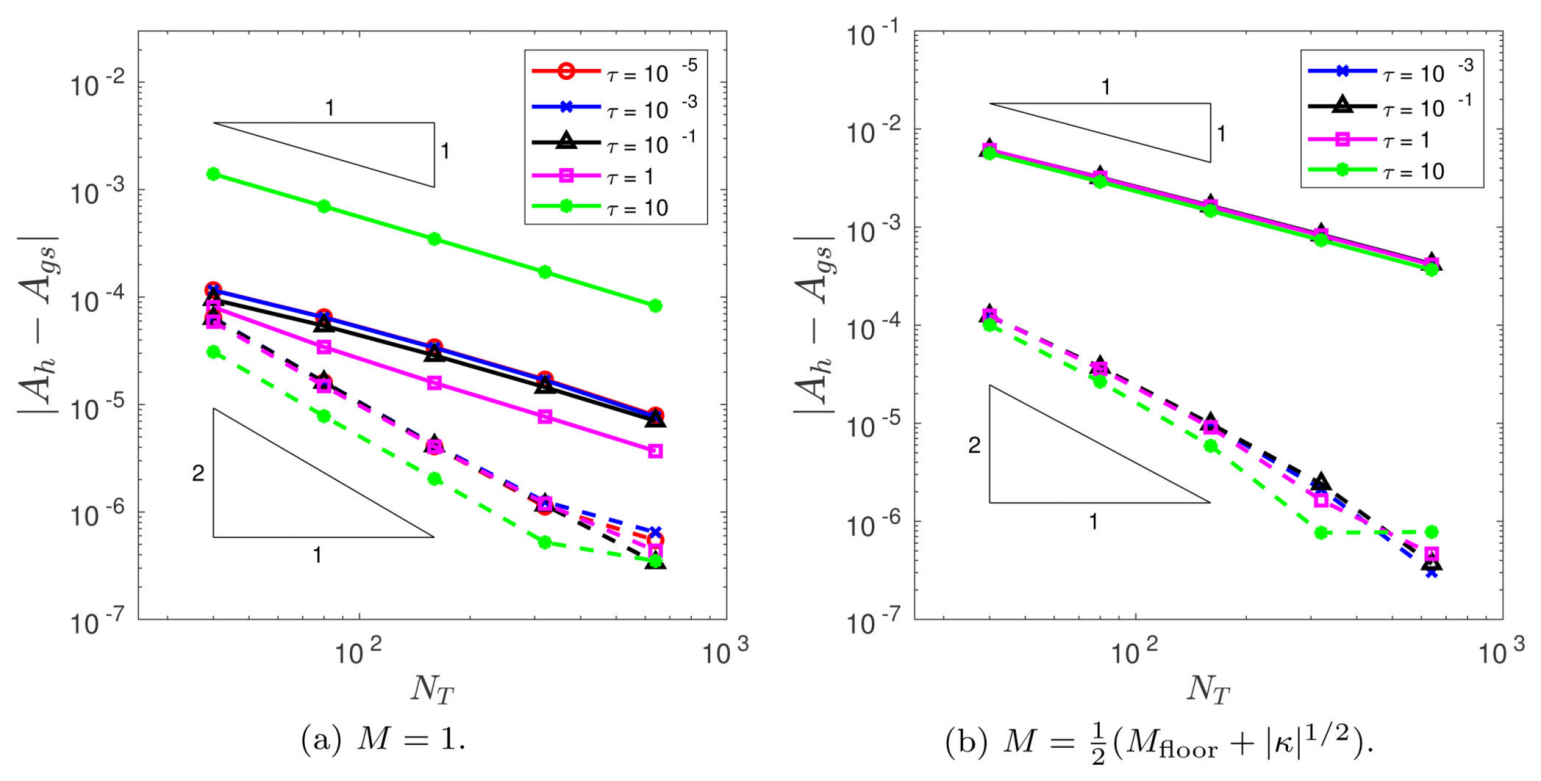
Temporal convergence in the absolute value error of the approximation of the enclosed area when the initial *l_p_*-ball is evolved by forced curve shortening flow using the BE (solid line) and CNBE (dashed line) schemes with *P* = *M*|***x***_*ξ*_|^2^ and (a) *M* = 1 or (b) $M=\frac{1}{2}\left(M_{\text{floor}}+|\kappa {|}^{1/2}\right).$

In all simulations, we run to a final time of *T* = 0.05. Once again, we assume a spatial balancing operator of the form *P* = *M*|***x**_*ξ*_*|^2^. The temporal convergence was tested for both the BE and CNBE schemes using a fine mesh resolution of *N* = 10^4^. Figure 13(a) illustrates the temporal convergence when *M* = 1. It is clear that the BE scheme demonstrates first-order convergence for all values of *τ*. For *τ* = 10, the error is significantly larger than for the other values of *τ*. The CNBE scheme demonstrates second-order convergence for all *τ*. Figure 13(b) illustrates the temporal convergence when $M=\frac{1}{2}\left(M_{\text{floor}}+{\left|\kappa \right|}^{1/2}\right)$. Once again, it is clear that the BE scheme demonstrates first-order convergence for all *τ* and, unlike when *M* = 1, the error value is similar for all *τ*. The CNBE scheme also once again demonstrates second-order convergence for all *τ* (except *τ* = 10^−5^, where the nonlinear Picard solver could not converge) and that the error values are similar.

Following the classical curve shortening examples (sections 4.1, 4.2, and 4.3), we wish to illustrate the computational efficiency of the nonlinear Picard solver. Table 8 displays the maximum and minimum number of Picard iterations required for each scheme and for each temporal resolution considered when *τ* = 1 for both monitor functions. It is clear that the computational cost of both the BE and CNBE schemes is small and that using a curvature-based monitor function does not substantially increase the computational cost of the Picard solver.

**Fig. 14:**
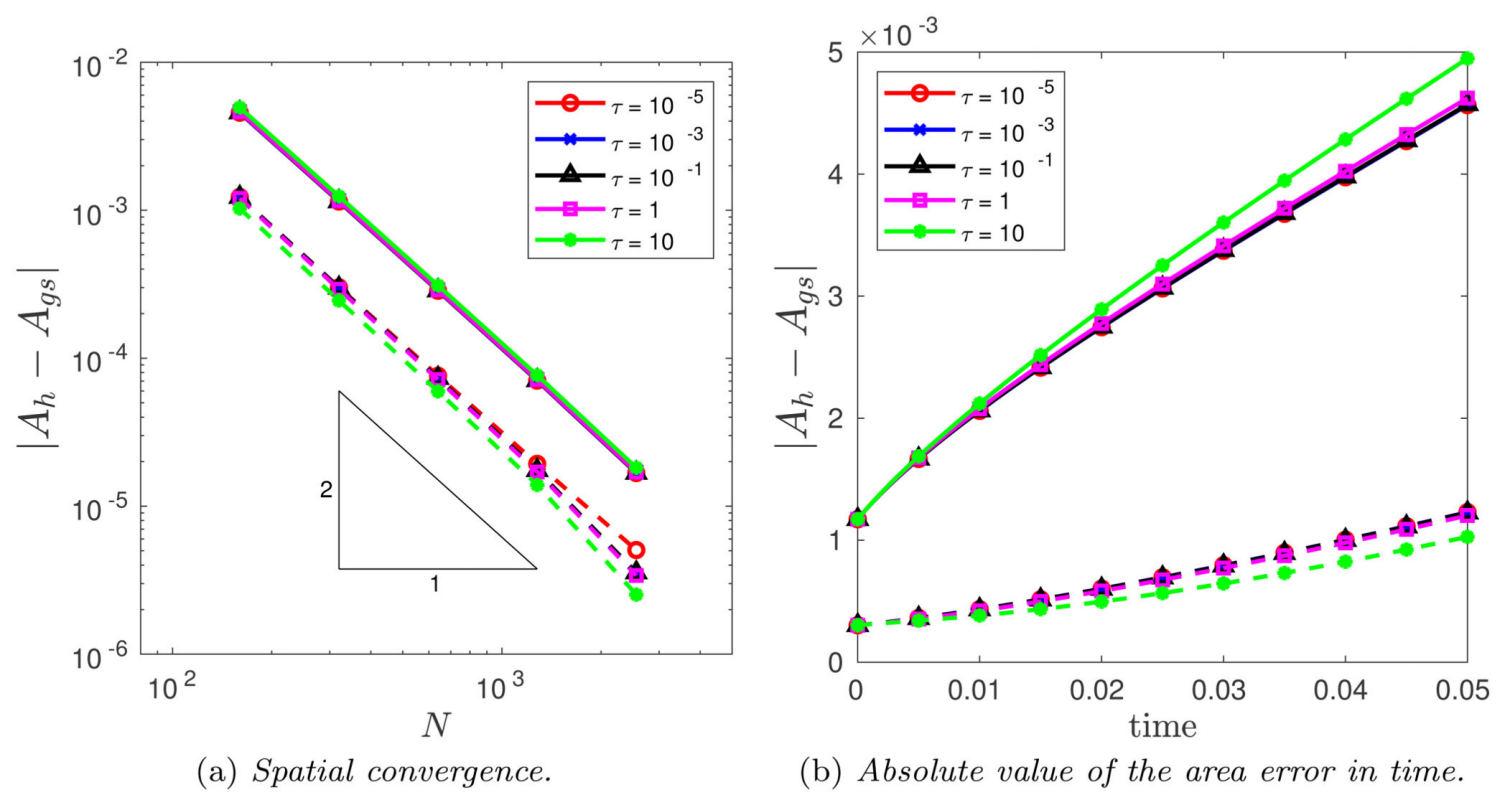
(a) Spatial convergence in the absolute value error of the approximation of the enclosed area when the initial *l_p_*-ball is evolved by forced curve shortening flow using the CNBE scheme for all *τ* and for both *M* = 1 (solid line) and $M=\frac{1}{2}\left(M_{\text{floor}}+|\kappa {|}^{1/2}\right)$ (dashed line). (b) Absolute value error in time for the CNBE scheme with *N* = 160 for both *M* = 1 (solid line) and $M=\frac{1}{2}\left(M_{\text{floor}}+|\kappa {|}^{1/2}\right)$ (dashed line).

The spatial convergence was tested using a large number of time steps, *N_T_* = 10^4^. Figure 14(a) illustrates the spatial convergence of the CNBE scheme when *M* = 1 (solid line) and $M=\frac{1}{2}\left(M_{\text{floor}}+{\left|\kappa \right|}^{1/2}\right)$ (dashed line). The convergence is clearly second-order for all values of *τ* for both *M* = 1 and $M=\frac{1}{2}\left(M_{\text{floor}}+{\left|\kappa \right|}^{1/2}\right)$. Crucially, once again the curvature-based monitor function produces an improved error compared with the uniform arc-length monitor function. As before, this is due to the curve being more accurately approximated using a curvature-based monitor function compared to uniform arc-length. To this end, Figure 14(b) illustrates the evolution of the absolute value error in time for the same spatial balancing operator for both *M* = 1 (solid line) and $M=\frac{1}{2}\left(M_{\text{floor}}+{\left|\kappa \right|}^{1/2}\right)$ (dashed line). Once again, it is clear that the curvature-based monitor function produces a much better mesh compared to uniform arc-length.

Figure 14(a) shows that, for a given monitor function, all values of *τ* perform similarly. Thus, for both monitor functions, Table 9 displays the maximum and minimum number of Picard iterations required for each scheme and for each spatial resolution considered when *τ* = 1. Once again, we observe that the computational cost of the nonlinear Picard solver is minimal, requiring at most two iterations per time step.

#### 4.5.2 Forced curve shortening flow of a nonconvex initial curve

In this section, the initial nonconvex curve is described parametrically as in (4.2). Figure 12(b) illustrates the initial (inner curve) and final (outer curve) mesh partitioning of the curve defined by (4.2) using *N* = 160 points. Once again, the initial mesh is obtained by equidistributing the curvature-based monitor function. This example was previously considered by Balažovjech and Mikula [2]. The final mesh position depicted in Figure 12(b) demonstrates good agreement with the final mesh position presented in [2] (their Figure 4.2). Note that the forcing constant used here (*β* = 10) is the same as that in [2].

**Fig. 15:**
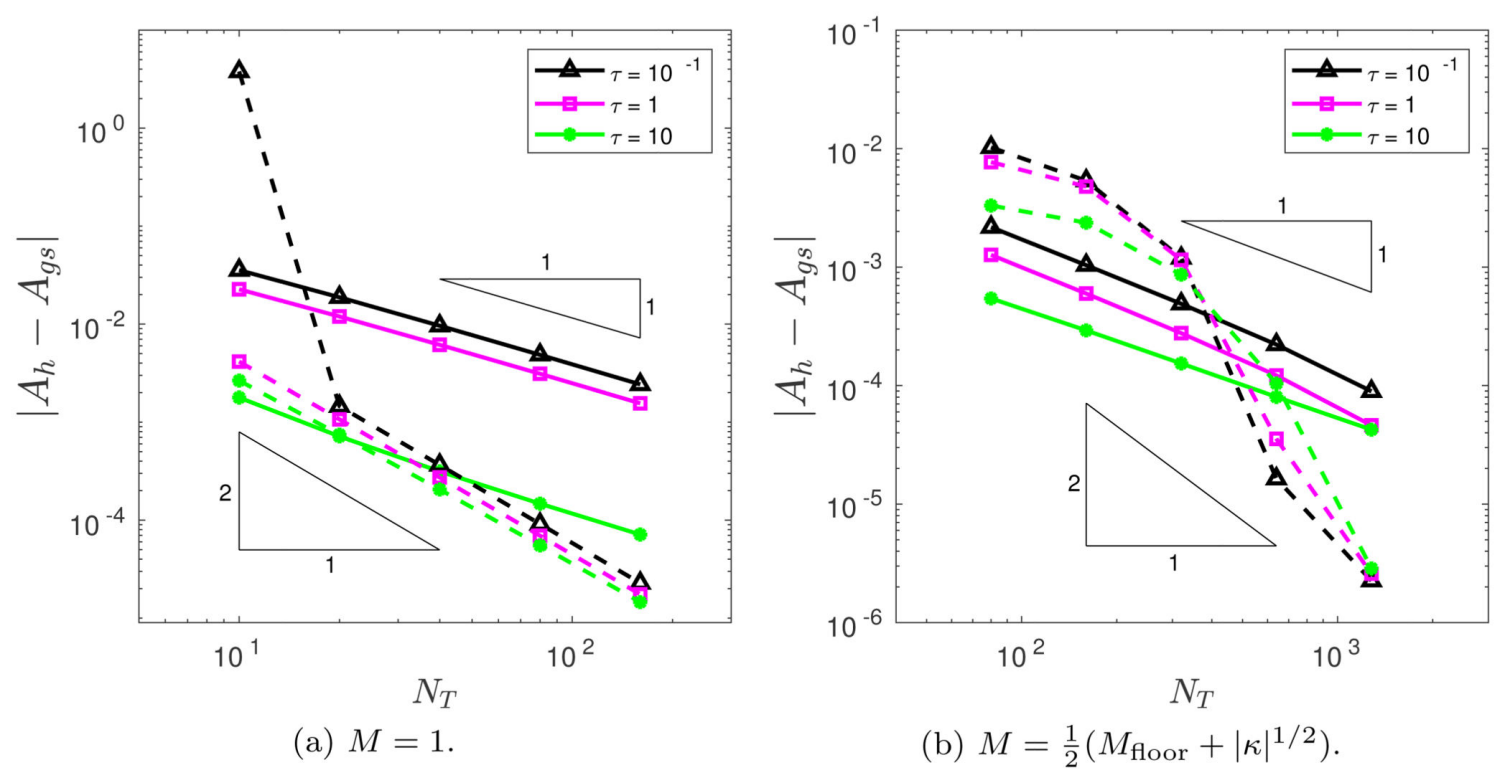
Temporal convergence in the absolute value error of the approximation of the enclosed area when the nonconvex initial curve is evolved by forced curve shortening flow using the BE (solid line) and CNBE (dashed line) schemes with *P* = *M*|***x***_*ξ*_|^2^ and (a) *M* = 1 or (b) $M=\frac{1}{2}\left(M_{\text{floor}}+|\kappa {|}^{1/2}\right).$

Following [2], all simulations ran to a final time of *T* = 0.02. Once again, we assume a spatial balancing operator of the form *P* = *M*|***x**_*ξ*_*|^2^. The temporal convergence was tested for both the BE and CNBE schemes using a fine mesh resolution of *N* = 10^4^. Figure 15(a) illustrates the temporal convergence when *M* = 1. No convergence results were obtained for either the BE or the CNBE scheme as the nonlinear Picard solver could not converge for *τ* = 10^−5^ and *τ* = 10^−3^. It is clear that the BE scheme demonstrates first-order convergence for all other values of *τ*. For *τ* = 10, the error is significantly lower than for the other values of *τ*. The CNBE scheme demonstrates second-order convergence for *τ* = 0.1, *τ* = 1, and *τ* = 10. Figure 13(b) illustrates the temporal convergence when $M=\frac{1}{2}\left(M_{\text{floor}}+{\left|\kappa \right|}^{1/2}\right)$. Once again, it is clear that for *τ* = 0.1, *τ* = 1, and *τ* = 10 the BE scheme demonstrates first-order convergence, while for the CNBE scheme, where the convergence rate is greater than second-order, we see the same accelerated convergence that was previously demonstrated (Figure 9(b)).

To illustrate the computational efficiency of the nonlinear Picard solver, we choose *τ* = 1. Tables 10 and 11 display the maximum and minimum number of Picard iterations required for each scheme and for each temporal resolution considered when *τ* = 1 for both monitor functions. For the smallest values of *N_T_* (Table 10) we see that when *M* = 1, both the BE and CNBE schemes struggle and require a large number of iterations per time step, but as *N_T_* is increased this number decreases. Table 10 also demonstrates an improvement in the maximum number of Picard iterations when the curvature-based monitor function is used. Indeed, this improvement is fairly substantial where a drop from 58 to 31 iterations can be observed for the CNBE scheme when *N_T_* = 10. Once again, as *N_T_* is increased, the maximum number of iterations decreases. For *N_T_* ≥ 320 (Table 11) the computational cost of the nonlinear Picard solver is once again low and continues to decrease as *N_T_* is increased for both schemes and for both monitor functions.

**Fig. 16:**
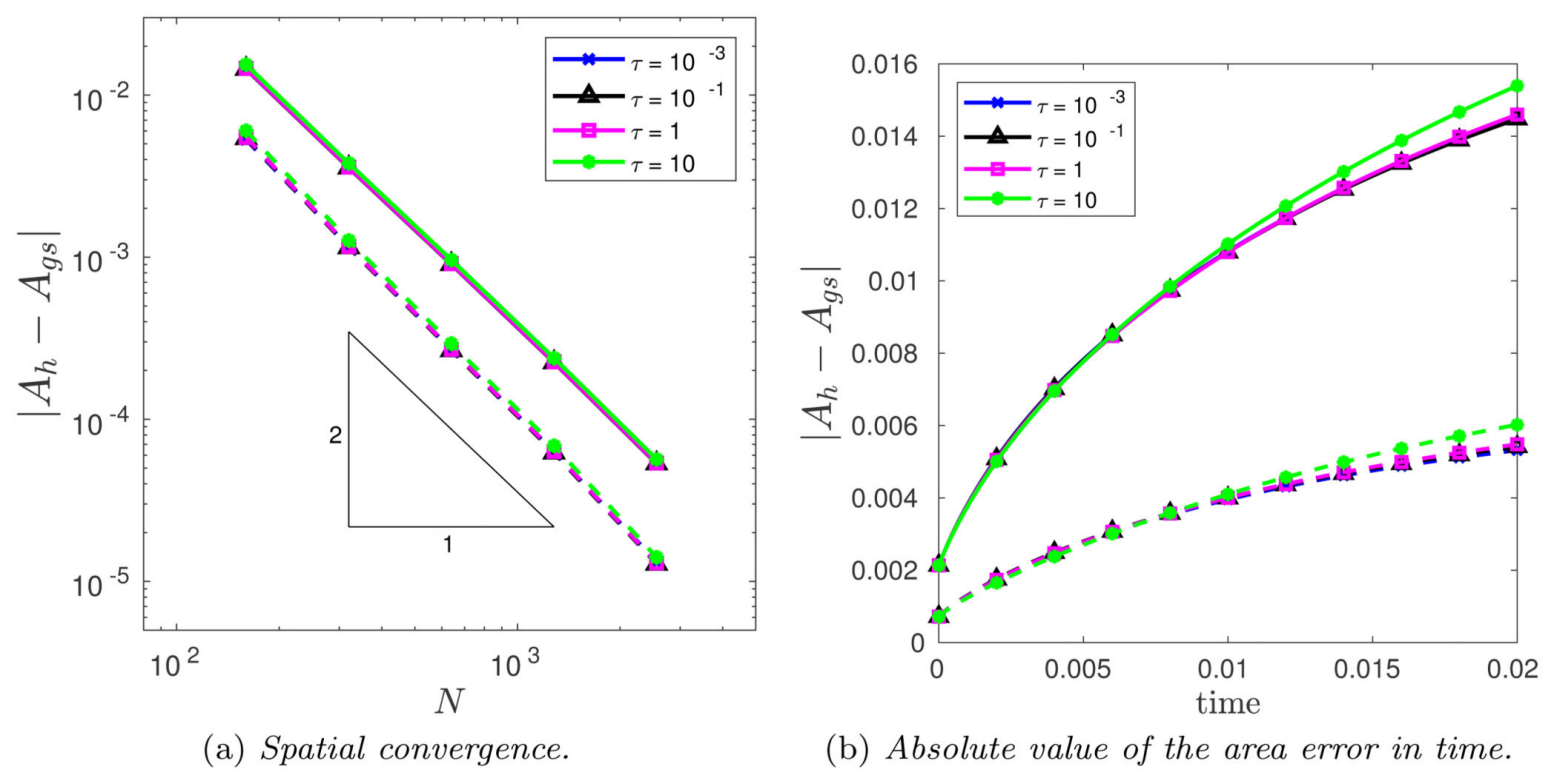
(a) Spatial convergence in the absolute value error of the approximation of the enclosed area when the nonconvex initial curve is evolved by forced curve shortening flow using the CNBE scheme for all *τ* and for both *M* = 1 (solid line) and $M=\frac{1}{2}\left(M_{\text{floor}}+|\kappa {|}^{1/2}\right)$ (dashed line). (b) Absolute value error in time for the CNBE scheme with *N* = 160 for both *M* = 1 (solid line) and $M=\frac{1}{2}\left(M_{\text{floor}}+|\kappa {|}^{1/2}\right)$ (dashed line).

The spatial convergence was tested using a large number of time steps *N_T_* = 10^4^. Figure 16(a) illustrates the spatial convergence of the CNBE scheme when *M* = 1 (solid line) and $M=\frac{1}{2}\left(M_{\text{floor}}+{\left|\kappa \right|}^{1/2}\right)$ (dashed line). The convergence is clearly second order for all values of *τ* for both *M* = 1 and $M=\frac{1}{2}\left(M_{\text{floor}}+{\left|\kappa \right|}^{1/2}\right)$. Crucially, once again the curvature-based monitor function produces an improved error compared to the uniform arc-length monitor function. Figure 16(b) illustrates the evolution of the absolute value error in time for the same spatial balancing operator for both *M* = 1 (solid line) and $M=\frac{1}{2}\left(M_{\text{floor}}+{\left|\kappa \right|}^{1/2}\right)$ (dashed line). Once again, it is clear that the curvature-based monitor function produces a much better mesh compared to uniform arc-length. Note that no convergence results were obtained for *τ* = 10^−5^.

Figure 16(a) shows that, for a given monitor function, all values of *τ* perform similarly. Thus, for both monitor functions, Table 12 displays the maximum and minimum number of Picard iterations required for each scheme and for each spatial resolution considered when *τ* = 1. Once again, we observe that the computational cost of the nonlinear Picard solver is minimal, requiring at most three iterations per time step.

## 5 Conclusions

We have presented an adaptive moving mesh method to simulate forced curve shortening flow. The method features a new strategy to control mesh movement in the tangential direction using a curvature-based monitor function. A novel hybrid time integration scheme has also been proposed. For classical and forced curve shortening flow of convex curves, the numerical experiments indicate that the method is spatially and temporally second-order accurate. We demonstrated the importance of the initial mesh for producing consistent convergence results (section 4.2.1) and presented a generalization of the de Boor algorithm that can be used to generate initially equidistributed grids (section 3.1).

For nonconvex curves evolved by classical and forced curve shortening flow, we found second-order temporal convergence when the uniform arc-length monitor function was used (Figures 9(a) and 15(a)) and at least second-order convergence when the curvature-based monitor function was used (Figures 9(b) and 15(b)). Analysis of this interesting observation is beyond the scope of this article and therefore is left as future work. Spatial second-order convergence was seen for classical and forced curve shortening of a nonconvex initial curve. Use of a curvature-based monitor function, has been shown to improve solution accuracy compared to the use of uniform arc-length meshes.

Our approach requires the solution of a nonlinear system, which we chose to obtain using Picard iterations. It was demonstrated that the computational cost of the nonlinear Picard solver is reasonable and that using a curvature-based monitor function did not significantly impact this cost. Here we enforced the nonlinear Picard solver to iterate to convergence (as was done in [3, 4]) and stopped the simulation when the solver was unable to converge. The lack of convergence in the Picard solver could be used as an indication of required temporal refinement. This interesting study of temporal adaptivity is beyond the scope of this article and therefore is also left as future work. For curve shortening flow with a singularity in finite time (section 4.4), we demonstrated that the lack of convergence of the nonlinear solver can be circumvented by fixing the maximum number of Picard iterations at a low value, thus allowing the numerics to *skip* the singularity, which is analogous to a semi-implicit approach [15].

Some immediate extensions include the application of the method to image segmentation problems and anisotropic curve evolution problems. The method can also be applied to physical or biological problems where the driving force of interface motion depends on field variables located on the interface. An important situation where this occurs is in the modeling of eukaryotic cell migration and chemotaxis, where the cell membrane is the interface between the extracellular and intracellular regions. Recent computational models assume that membrane motion is driven by mechanical and biochemical forces which depend on the cell receptor and protein densities on the membrane as well as the membrane curvature [28, 27, 29, 21, 22]. For all of these problems it is possible to use the adaptive moving mesh approach proposed here to resolve solution features along the interface. It remains to be seen how to devise a suitable monitor function to redistribute mesh points to simultaneously resolve interface geometry and interface-bound solution fields.

In the future we also plan to extend the adaptive moving mesh technique to the evolution of surfaces in three dimensions. For this class of problems it is especially important to devise a tangential velocity field to avoid problems with degenerating grid quality. Several methods have recently been proposed based on the control of volume, angle, and length metrics [23, 26]; discrete conformal mappings [4]; and harmonic mappings [15]. Some work has also been done on the use of moving mesh methods for stationary surfaces, including a sphere [13, 34, 35], and parametric surfaces [9]. However, none of these methods has been specifically devised to include solution adaptivity on evolving surfaces. It is hoped that the experience of applying moving mesh methods for two-dimensional planar problems [7] can be used to develop robust and adaptive surface evolution techniques to be applied to the solutions of PDEs on evolving surfaces.

## Figures and Tables

**Table 1:** (Circle.) Maximum and minimum number of Picard steps required for each scheme, with *N* = 10^4^, for each temporal resolution.

	*N_T_* = 10		*N_T_* = 20		*N_T_* = 40		*N_T_* = 80		*N_T_* = 160	
	Max	Min	Max	Min	Max	Min	Max	Min	Max	Min
BE	6	5	5	4	4	3	3	3	3	3
CNBE	5	5	4	4	4	3	3	3	3	3

**Table 2:** (Circle.) Maximum and minimum number of Picard steps required for each scheme, with *N_T_* = 10^4^, for each spatial resolution.

	*N* = 160		*N* = 320		*N* = 640		*N* = 1280		*N* = 2560	
	Max	Min	Max	Min	Max	Min	Max	Min	Max	Min
BE	2	2	2	2	2	2	2	2	2	2
CNBE	2	2	2	2	2	2	2	2	2	2

**Table 3:** (Ellipse.) Maximum and minimum number of Picard steps required for each scheme, with *N* = 10^3^, for each temporal resolution when *τ* = 10 and *P* = *M*|***x***_*ξ*_|^2^, for both *M* = 1 and $M=\frac{1}{2}\left(M_{\text{floor}}+{\left|\kappa \right|}^{1/2}\right).$

	*M* = 1									
	*N_T_* = 10		*N_T_* = 20		*N_T_* = 40		*N_T_* = 80		*N_T_* = 160	
	Max	Min	Max	Min	Max	Min	Max	Min	Max	Min
BE	23	10	23	8	13	7	8	5	7	4
CNBE	20	9	11	7	9	6	8	5	7	4

	$M=\frac{1}{2}\left(M_{\text{floor}}+{\left|\kappa \right|}^{1/2}\right)$									
	*N_T_* = 10		*N_T_* = 20		*N_T_* = 40		*N_T_* = 80		*N_T_* = 160	
	Max	Min	Max	Min	Max	Min	Max	Min	Max	Min
BE	22	9	33	7	12	6	7	4	6	4
CNBE	20	9	11	7	7	5	6	4	6	4

**Table 4:** (Ellipse.) Maximum and minimum number of Picard steps required for each scheme, with N_T_ = 10^4^, for each spatial resolution when τ = 10 and $P=M{\left|{\text{x}}_{\xi }\right|}^{2},$ for both *M* = 1 and $M=\frac{1}{2}\left(M_{\text{floor}}+{\left|\kappa \right|}^{1/2}\right).$

	*M* = 1									
	*N* = 160		*N* = 320		*N* = 640		*N* = 1280		*N* = 2560	
	Max	Min	Max	Min	Max	Min	Max	Min	Max	Min
BE	3	2	3	2	3	2	3	2	3	2
CNBE	3	2	3	2	3	2	3	2	3	2

	$M=\frac{1}{2}\left(M_{\text{floor}}+{\left|\kappa \right|}^{1/2}\right)$									
	*N* = 160		*N* = 320		*N* = 640		*N* = 1280		*N* = 2560	
	Max	Min	Max	Min	Max	Min	Max	Min	Max	Min
BE	3	2	3	2	3	2	3	2	3	2
CNBE	2	2	2	2	2	2	2	2	2	2

**Table 5:** (Nonconvex.) Maximum and minimum number of Picard steps required for each scheme, with *N* = 10^3^, for each temporal resolution *N_T_* = 10, 20, 40, 80, 160 when *τ* = 10 and $P=M{\left|{\text{x}}_{\xi }\right|}^{2},$ for both *M* = 1 and $M=\frac{1}{2}\left(M_{\text{floor}}+{\left|\kappa \right|}^{1/2}\right).$

	*M* = 1									
	*N_T_* = 10		*N_T_* = 20		*N_T_* = 40		*N_T_* = 80		*N_T_* = 160	
	Max	Min	Max	Min	Max	Min	Max	Min	Max	Min
BE	21	8	18	7	17	5	18	5	16	4
CNBE	29	7	16	6	14	5	14	4	13	4

	$M=\frac{1}{2}\left(M_{\text{floor}}+{\left|\kappa \right|}^{1/2}\right)$									
	*N_T_* = 10		*N_T_* = 20		*N_T_* = 40		*N_T_* = 80		*N_T_* = 160	
	Max	Min	Max	Min	Max	Min	Max	Min	Max	Min
BE	14	9	13	7	11	6	11	5	11	4
CNBE	26	10	17	8	12	7	12	5	10	4

**Table 6:** (Nonconvex.) Maximum and minimum number of Picard steps required for each scheme, with *N* = 10^3^, for each temporal resolution *N_T_* = 320, 640, 1280, 2560 when *τ* = 10 and *P* = *M*|***x***_*ξ*_|^2^, for both *M* = 1 and $M=\frac{1}{2}\left(M_{\text{floor}}+{\left|\kappa \right|}^{1/2}\right).$

	*M* = 1							
	*N_T_* = 320		*N_T_* = 640		*N_T_* = 1280		*N_T_* = 2560	
	Max	Min	Max	Min	Max	Min	Max	Min
BE	14	3	11	3	8	3	7	2
CNBE	11	3	9	3	8	3	6	2

	$M=\frac{1}{2}\left(M_{\text{floor}}+{\left|\kappa \right|}^{1/2}\right)$									
	*N_T_* = 320		*N_T_* = 640		*N_T_* = 1280		*N_T_* = 2560	
	Max	Min	Max	Min	Max	Min	Max	Min
BE	11	3	9	3	7	3	6	2
CNBE	10	3	9	3	8	3	6	2

**Table 7:** (Nonconvex.) Maximum and minimum number of Picard steps required for each scheme, with *N_T_* = 10^4^, for each spatial resolution when *τ* = 1 and *P* = *M*|***x***_*ξ*_|^2^, for both *M* = 1 and $M=\frac{1}{2}\left(M_{\text{floor}}+{\left|\kappa \right|}^{1/2}\right).$

	*M* = 1									
	*N* = 160		*N* = 320		*N* = 640		*N* = 1280		*N* = 2560	
	Max	Min	Max	Min	Max	Min	Max	Min	Max	Min
BE	5	2	6	2	6	2	6	2	7	2
CNBE	5	2	6	2	6	2	6	2	6	2

	$M=\frac{1}{2}\left(M_{\text{floor}}+{\left|\kappa \right|}^{1/2}\right)$									
	*N* = 160		*N* = 320		*N* = 640		*N* = 1280		*N* = 2560	
	Max	Min	Max	Min	Max	Min	Max	Min	Max	Min
BE	4	2	5	2	5	2	5	2	5	2
CNBE	4	2	5	2	5	2	5	2	5	2

**Table 8:** (Forced *l_p_*-ball.) Maximum and minimum number of Picard steps required for each scheme, with *N* = 10^4^, for each temporal resolution *N_T_* = 40, 80, 160, 320, 640 when *τ* = 1 and $P=M{\left|{\text{x}}_{\xi }\right|}^{2},$ for both *M* = 1 and $M=\frac{1}{2}\left(M_{\text{floor}}+{\left|\kappa \right|}^{1/2}\right).$

	*M* = 1									
	*N_T_* = 40		*N_T_* = 80		*N_T_* = 160		*N_T_* = 320		*N_T_* = 640	
	Max	Min	Max	Min	Max	Min	Max	Min	Max	Min
BE	6	4	5	4	5	3	4	3	3	3
CNBE	6	4	5	4	5	3	4	3	3	3

	$M=\frac{1}{2}\left(M_{\text{floor}}+{\left|\kappa \right|}^{1/2}\right)$									
	*N_T_* = 40		*N_T_* = 80		*N_T_* = 160		*N_T_* = 320		*N_T_* = 640	
	Max	Min	Max	Min	Max	Min	Max	Min	Max	Min
BE	7	5	6	4	5	4	4	3	3	3
CNBE	7	5	6	4	5	4	4	3	3	3

**Table 9:** (Forced *l_p_*-ball.) Maximum and minimum number of Picard steps required for each scheme, with *N_T_* = 10^4^, for each spatial resolution when *τ* = 1 and $P=M{\left|{\text{x}}_{\xi }\right|}^{2},$ for both *M* = 1 and $M=\frac{1}{2}\left(M_{\text{floor}}+{\left|\kappa \right|}^{1/2}\right).$

	*M* = 1									
	*N* = 160		*N* = 320		*N* = 640		*N* = 1280		*N* = 2560	
	Max	Min	Max	Min	Max	Min	Max	Min	Max	Min
BE	2	2	2	2	2	2	2	2	2	2
CNBE	2	2	2	2	2	2	2	2	2	2

	$M=\frac{1}{2}\left(M_{\text{floor}}+{\left|\kappa \right|}^{1/2}\right)$									
	*N* = 160		*N* = 320		*N* = 640		*N* = 1280		*N* = 2560	
	Max	Min	Max	Min	Max	Min	Max	Min	Max	Min
BE	2	2	2	2	2	2	2	2	2	2
CNBE	2	2	2	2	2	2	2	2	2	2

**Table 10:** (Forced nonconvex.) Maximum and minimum number of Picard steps required for each scheme, with *N* = 10^4^, for each temporal resolution *N_T_* = 10, 20, 40, 80, 160 when *τ* = 1 and $P=M{\left|{\text{x}}_{\xi }\right|}^{2},$ for both *M* = 1 and $M=\frac{1}{2}\left(M_{\text{floor}}+{\left|\kappa \right|}^{1/2}\right).$

	*M* = 1									
	*N_T_* = 10		*N_T_* = 20		*N_T_* = 40		*N_T_* = 80		*N_T_* = 160	
	Max	Min	Max	Min	Max	Min	Max	Min	Max	Min
BE	51	18	45	13	35	9	25	7	17	5
CNBE	58	20	45	14	34	10	24	7	17	5

	$M=\frac{1}{2}\left(M_{\text{floor}}+{\left|\kappa \right|}^{1/2}\right)$									
	*N_T_* = 10		*N_T_* = 20		*N_T_* = 40		*N_T_* = 80		*N_T_* = 160	
	Max	Min	Max	Min	Max	Min	Max	Min	Max	Min
BE	30	14	23	10	16	8	12	6	9	6
CNBE	31	16	23	9	17	8	12	7	9	6

**Table 11:** (Forced nonconvex.) Maximum and minimum number of Picard steps required for each scheme, with *N* = 10^4^, for each temporal resolution *N_T_* = 320, 640, 1280, 2560 when *τ* = 1 and $P=M{\left|{\text{x}}_{\xi }\right|}^{2},$ for both *M* = 1 and $M=\frac{1}{2}\left(M_{\text{floor}}+{\left|\kappa \right|}^{1/2}\right).$

	*M* = 1							
	*N_T_* = 320		*N_T_* = 640		*N_T_* = 1280		*N_T_* = 2560	
	Max	Min	Max	Min	Max	Min	Max	Min
BE	12	4	8	3	6	3	5	2
CNBE	12	4	8	3	6	3	4	2

	$M=\frac{1}{2}\left(M_{\text{floor}}+{\left|\kappa \right|}^{1/2}\right)$									
	*N_T_* = 320		*N_T_* = 640		*N_T_* = 1280		*N_T_* = 2560	
	Max	Min	Max	Min	Max	Min	Max	Min
BE	8	5	6	5	6	4	5	4
CNBE	7	6	7	5	6	4	5	4

**Table 12:** (Forced nonconvex.) Maximum and minimum number of Picard steps required for each scheme, with *N_T_* = 10^4^, for each spatial resolution when *τ* = 1 and $P=M{\left|{\text{x}}_{\xi }\right|}^{2},$ for both *M* = 1 and $M=\frac{1}{2}\left(M_{\text{floor}}+{\left|\kappa \right|}^{1/2}\right).$

	*M* = 1									
	*N* = 160		*N* = 320		*N* = 640		*N* = 1280		*N* = 2560	
	Max	Min	Max	Min	Max	Min	Max	Min	Max	Min
BE	3	2	3	2	3	2	3	2	3	2
CNBE	3	2	3	2	3	2	3	2	3	2

	$M=\frac{1}{2}\left(M_{\text{floor}}+{\left|\kappa \right|}^{1/2}\right)$									
	*N* = 160		*N* = 320		*N* = 640		*N* = 1280		*N* = 2560	
	Max	Min	Max	Min	Max	Min	Max	Min	Max	Min
BE	2	2	3	2	3	2	3	2	3	2
CNBE	2	2	3	2	3	2	3	2	3	2
